# Sustainable Porous Carbon Derived from Lignin for High‐Performance CO_2_ Capture

**DOI:** 10.1002/asia.202500988

**Published:** 2026-02-23

**Authors:** Kiet Le Anh Cao, Oktaviardi Bityasmawan Abdillah, Tomoyuki Hirano, Eka Lutfi Septiani, Takashi Ogi

**Affiliations:** ^1^ Chemical Engineering Program, Department of Advanced Science and Engineering, Graduate School of Advanced Science and Engineering Hiroshima University Higashi‐Hiroshima Hiroshima Japan

**Keywords:** CO_2_ capture, lignin, nanostructured material, porous carbon, sustainable synthesis

## Abstract

The accelerating rise of atmospheric CO_2_ remains a central driver of global climate change, highlighting the urgent need for scalable and energy‐efficient carbon capture technologies. Porous carbons are among the most promising solid adsorbents due to their high surface area, chemical stability, and tunable pore structures, which facilitate efficient CO_2_ adsorption and low regeneration energy. Lignin is a renewable aromatic by‐product of the pulp and paper industry, which offers exceptional promise as a sustainable carbon source due to its abundance (50–70 Mt/year), high carbon content (>60 wt%), and rich aromatic structure. Unlike previous reviews broadly covering biomass‐derived carbons, this review focuses on recent advances in lignin‐derived porous carbons for CO_2_ capture, correlating preparation strategies with structural evolution and adsorption performance. Chemical activation, templating, and hybrid methods enable precise control of ultramicropores (<0.7 nm), mesoporous channels, and heteroatom functionalities, which synergistically determine adsorption capacity, selectivity, and regeneration energy. Emerging approaches such as amine functionalization introduce strong chemisorption sites for post‐combustion and direct‐air capture, while AI‐assisted design accelerates understanding of synthesis–property–performance relationships. Despite remarkable progress, remaining challenges in feedstock variability, scalability, and greener process development are discussed along with future prospects for sustainable CO_2_ capture using lignin‐derived porous carbons.

## Graphical abstract

Lignin‐derived porous carbons offer a sustainable and scalable route for high‐performance CO_2_ capture. By tailoring ultramicroporosity, hierarchical pore networks, and heteroatom functionalities through chemical activation, templating, and hybrid methods, adsorption capacity and selectivity can be optimized. This review correlates. synthesis–structure–performance relationships to enable the rational design of scalable, energy‐efficient carbon capture materials for circular carbon technologies.

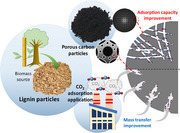

## 1 Introduction

Addressing climate change is one of the most urgent challenges of our time, demanding coordinated scientific, technological, and policy efforts. Global warming, primarily driven by rising concentrations of greenhouse gases in the atmosphere, has led to widespread environmental, economic, and societal impacts, including disruption of ecosystems, threats to food and water security, and increased human health risks [1, 2]. Among the major greenhouse gases, carbon dioxide (CO_2_) is the predominant contributor due to its long atmospheric lifetime and direct correlation with fossil fuel combustion and industrial activity [3]. Since the onset of industrialization, atmospheric CO_2_ concentrations have risen from approximately 280 ppm to over 420 ppm, and this upward trend continues despite global mitigation efforts. Although renewable energy technologies are advancing rapidly, the complete replacement of fossil‐based energy systems remains a long‐term goal. Therefore, scalable and energy‐efficient CO_2_ mitigation strategies remain critical. Key technological pathways include carbon capture and sequestration (CCS), carbon capture and utilization (CCU), and reactive carbon capture (RCC), which are essential for managing emissions from CO_2_‐intensive industries such as power generation, cement, and steel production [4, 5, 6].

Current CO_2_ capture technologies include post‐combustion, pre‐combustion, and oxy‐fuel combustion processes, each requiring tailored separation strategies [7, 8]. Amine‐based liquid solvents remain the most established method for post‐combustion CO_2_ capture. However, issues related to energy‐intensive regeneration, solvent degradation, and high operational costs significantly limit their widespread adoption [9]. As an alternative, porous solid adsorbents have emerged as promising materials due to their lower regeneration energy requirements, thermal and chemical stability, and compatibility with modular capture systems [10, 11, 12]. Porous solids, including zeolites [13], metal‐organic frameworks [14], covalent organic frameworks [15], silica [16], and porous carbons [17], offer high specific surface areas and tunable pore structures that enable efficient CO_2_ physisorption. However, many of these materials suffer from drawbacks such as high production costs, sensitivity to moisture, and limited scalability. In contrast, porous carbon materials have attracted significant interest due to their chemical stability, cost‐effectiveness, and the possibility of tailoring their textural and surface properties [18, 19, 20, 21]. Notably, porous carbons can be derived from a broad range of biomass feedstocks, including agricultural residues, industrial by‐products, and food waste, offering a sustainable route to high‐performance sorbents. Among various biomass sources, lignin stands out as a particularly attractive precursor for porous carbon production [22, 23, 24]. As the second most abundant natural polymer and the only one rich in aromatic content, lignin accounts for approximately 10–35 wt% of plant biomass [25]. Lignin is primarily obtained as a by‐product from the pulp and paper industry and emerging biorefinery processes, yet it remains largely underutilized and is typically burned for low‐grade heat recovery [26]. This underuse represents a significant missed opportunity, as lignin offers the highest carbon yield among biopolymers and possesses a structurally versatile aromatic backbone suitable for physical and chemical modification.

Recent studies have shown that lignin‐derived porous carbon can be engineered with high specific surface area, tunable porosity, and tailored surface functionalities (e.g., oxygen or nitrogen groups) favorable for selective CO_2_ adsorption [27, 28, 29]. These properties can be enhanced through controlled carbonization, physical/chemical activation, and functionalization processes. Furthermore, the conversion of lignin into porous carbon aligns with the principles of green chemistry and carbon circularity, simultaneously enabling the valorization of industrial waste and the development of materials that contribute to climate change mitigation. Importantly, lignin‐based carbons can be tuned to achieve moderate adsorption enthalpies (typically 20–35 kJ/mol), as CO_2_ is mainly bound through physical interactions within micropores rather than strong chemical bonding, thereby improving energy efficiency during regeneration cycles, which is an essential advantage for practical carbon capture applications [30]. Nevertheless, the full industrial potential of lignin‐derived porous carbon remains unrealized, primarily due to variability in lignin composition arising from different plant sources, pulping processes, and extraction methods, which leads to inconsistent carbon structure and performance. This variability, combined with the need for scalable, cost‐effective, and environmentally benign activation techniques, continues to impede commercialization. As such, advancing scalable processes that accommodate feedstock heterogeneity while delivering reproducible performance is a critical focus for future research and technological development.

**FIGURE 1:**
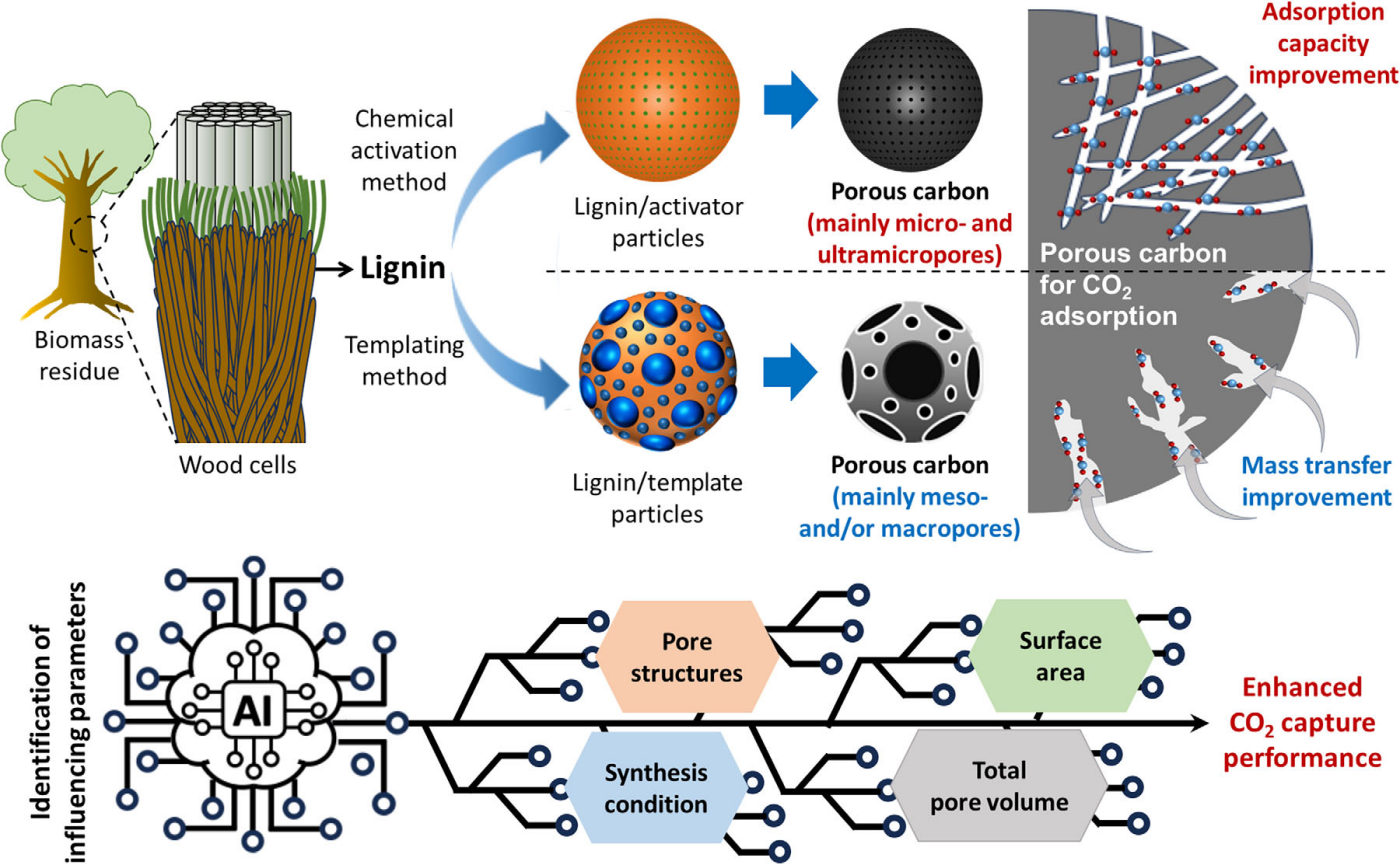
Schematic illustration of the conversion of lignin into porous carbon materials for high‐efficiency CO_2_ capture.

In this review, we present a comprehensive overview of recent developments in the preparation of sustainable porous carbon derived from lignin for high‐performance CO_2_ capture, with a focus on addressing several unresolved challenges that hinder commercialization. Despite significant research activity, key issues remain including (i) poor control over pore size distribution that limits adsorption performance, (ii) limited understanding of structure‐property‐performance relationships that govern CO_2_ adsorption capacity and selectivity, and (iii) the lack of scalable, green, and economically viable activation and functionalization methods. Previous reviews have either focused broadly on biomass‐derived carbon or treated lignin as a minor component [31, 32, 33]. In contrast, this review uniquely concentrates on lignin as a standalone precursor and connects recent synthesis strategies with the resulting material properties and their CO_2_ adsorption behavior. Figure 1 provides an overview of the conversion of lignin into porous carbon materials designed for efficient CO_2_ capture. The scheme illustrates how different synthesis routes (e.g., chemical activation, templating methods) tailor the resulting pore architecture and surface chemistry. Particular attention is given to the formation of micro‐, meso‐, and macroporous networks, where micropores (especially ultramicropores) primarily govern CO_2_ adsorption capacity and meso‐/macropores enhance diffusion and accessibility. The key concepts and synthesis–structure–performance relationships depicted in Figure 1 are discussed in detail in the following sections, providing a comprehensive understanding of how lignin‐derived porous carbons can be engineered for sustainable and high‐efficiency CO_2_ capture. Because these relationships involve multiple interdependent variables that are difficult to optimize simultaneously using conventional trial‐and‐error approaches, an emerging strategy such as artificial intelligence (AI)‐guided material design is introduced as a powerful tool to navigate this complex design space. Finally, we identify current limitations, highlight innovative approaches, and propose future research directions to overcome current barriers and enable the practical deployment of lignin‐derived porous carbons, contributing to the development of sustainable, circular carbon capture technologies.

## 2 Lignin Chemistry and Properties

### 2.1 Lignin and Green Chemistry

**FIGURE 2:**
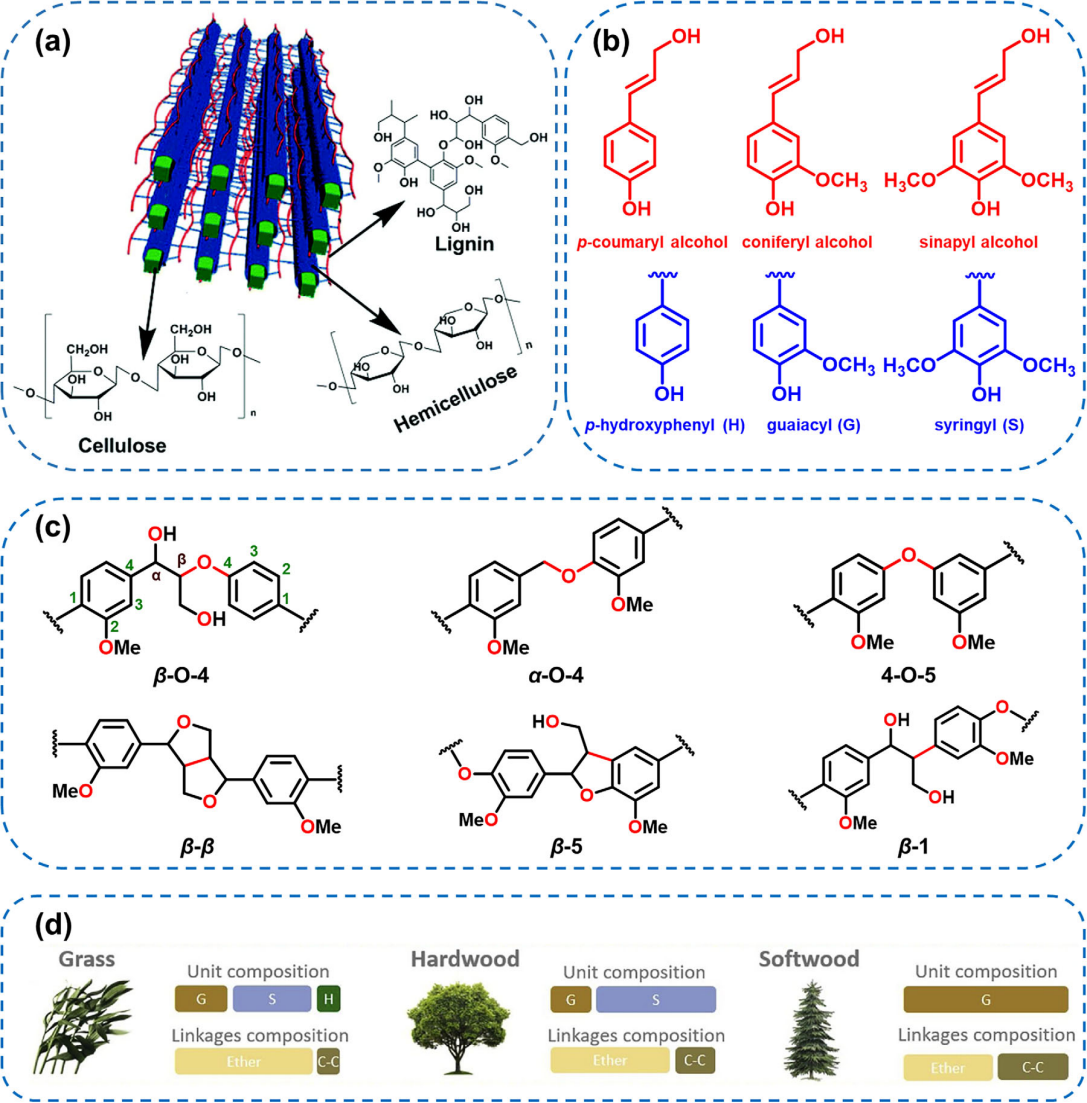
(a) Schematic analysis of the location and structure of lignin in a lignocellulosic biomass. Reproduced with permission [46]. (b) Basic building blocks of lignin (monolignol) and their corresponding structural polymers, (c) the typical linkages of lignin, and (d) the content of monomers and linkages in lignin from various sources. Reproduced with permission [45].

The shift toward sustainable production of energy, fuels, and chemicals from renewable sources remains a pressing challenge in the global pursuit of a low‐carbon economy. Among the various renewable feedstocks, lignocellulosic biomass, comprising hardwoods, softwoods, and grasses, stands out due to its abundance and rich composition of cellulose, hemicellulose, and lignin (Figure 2a) [25]. This biomass offers a viable alternative to fossil‐derived resources for the manufacture of biofuels and bio‐based chemicals. In most biorefinery operations, the primary focus has traditionally been on the conversion of cellulose into fermentable sugars for ethanol production or platform chemicals. However, these processes often leave behind lignin as a complex byproduct, typically in a degraded form with limited utilization. Similarly, the pulp and paper industry generates large amounts of technical lignin as a by‐product of cellulose extraction, most of which are incinerated for energy recovery, thereby underutilizing their full chemical potential.

Lignin, as a renewable biopolymer enriched with aromatic functionalities, holds tremendous promise for value‐added applications across multiple industries [34]. Broadly, lignin valorization has been explored through three main routes: (i) conversion into energy carriers such as bio‐oil, syngas, or green fuels; (ii) utilization as a macromolecular material in composites or structural applications; and (iii) depolymerization into aromatic monomers that serve as building blocks for fine and bulk chemicals [35]. These aromatic building blocks are highly relevant for the synthesis of complex heterocyclic frameworks used in pharmaceuticals and functional molecules [36]. Notably, recent advances in lignin chemistry have accelerated efforts to transform lignin into high‐value products such as carbon fibers, polyurethane foams, adhesives, emulsifiers, dispersants, thermoplastics, polymer additives, and even epoxy resins for electronic devices [37]. In addition, lignin has recently gained attention as a sustainable precursor for porous carbon materials, which exhibit high surface area, tunable pore structure, and excellent chemical stability, making them promising candidates for applications in CO_2_ capture [28, 38, 39], supercapacitors [23, 24, 40, 41], and catalysis [42, 43].

Within integrated biorefineries, where all biomass fractions (cellulose, hemicellulose, and lignin) are efficiently utilized, there is growing emphasis on developing economically viable and environmentally responsible strategies for lignin upgrading. Rather than treating lignin solely as a low‐value energy source, emerging green chemistry approaches aim to unlock its potential as a feedstock for producing bio‐based aromatic compounds. The inherent polyaromatic structure of lignin enables the generation of a diverse range of value‐added chemicals, polymers, and specialty materials with potential applications in sectors such as coatings, plastics, packaging, and household products. These compounds span a broad range of markets and economic value, positioning lignin as a key renewable source of aromatics in the circular bioeconomy. Importantly, the transition toward lignin‐based value chains must align with the principles of green chemistry, favoring low‐toxicity reagents, energy‐efficient processes, and waste minimization. The development of clean, catalytic, and selective methods for lignin transformation not only enhances its economic attractiveness but also reinforces the sustainability of second‐generation biorefineries. Exploring novel lignin‐derived products and expanding into new application markets are critical steps toward establishing a circular and environmentally friendly chemical industry.

### 2.2 Composition and Structure of Lignin

In the plant cell wall, lignin plays a critical structural role by occupying the interstitial spaces between cellulose and hemicellulose. Acting as a natural adhesive, it cross‐links with these carbohydrate polymers, thereby enhancing the mechanical strength, rigidity, and hydrophobicity of the lignocellulosic matrix. Chemically, lignin is a highly complex, three‐dimensional amorphous polymer built from methoxylated phenylpropanoid units, making it the most abundant renewable source of aromatic carbon in nature [44]. The composition and content of lignin vary significantly depending on plant type, species, and even specific anatomical regions within a single plant. Typically, lignin constitutes around 30% of the dry weight in softwoods, 20%–25% in hardwoods, and about 10%–15% in grasses. These differences reflect both the evolutionary adaptations of plant species and the underlying biochemical pathways regulating lignin biosynthesis.

Lignin is biosynthesized via the phenylpropanoid pathway, where three primary monolignols (*p*‐coumaryl alcohol, coniferyl alcohol, and sinapyl alcohol) undergo oxidative radical polymerization catalyzed by enzymes such as laccases and peroxidases [45]. These monolignols are incorporated into the lignin macromolecule as *p*‐hydroxyphenyl (H), guaiacyl (G), and syringyl (S) units, respectively (Figure 2b). Each unit consists of a phenylpropane backbone but differs in the number of methoxy substituents on the aromatic ring: none for H, one for G, and two for S. The relative abundance of these units defines the type of lignin: softwoods predominantly contain G‐type lignin, hardwoods are rich in both G and S units (GS‐type), and grasses feature all three types (HGS‐type) (Figure 2d).

The diversity in monomeric composition and the various inter‐unit linkages, such as β–O–4 (aryl ether), β–β (resinol), and β–5 (phenylcoumaran) bonds, make lignin structurally heterogeneous and chemically recalcitrant (Figure 2c) [25]. Unlike other natural biopolymers, lignin cannot be isolated in its native, unmodified form due to its tight integration within the plant cell wall and susceptibility to alteration during extraction. Consequently, our current understanding of its structure is primarily based on studies of isolated lignin fractions, including milled wood lignin, dioxane lignin, and industrial lignin such as kraft lignin. Moreover, in addition to the canonical H, G, and S units, lignin may also incorporate various non‐traditional subunits and functional moieties. These include ferulic acid esters, which create cross‐links between hemicellulose and lignin, as well as other intermediates like coniferaldehyde, sinapaldehyde, 5‐hydroxyconiferyl alcohol, and acylated monolignols bearing side groups such as acetate, *p*‐coumarate, or *p*‐hydroxybenzoate. These structural variations further complicate lignin's architecture but also present unique opportunities for targeted chemical modifications and material development.

Understanding the fundamental chemistry and variability of lignin is essential for tailoring its transformation into high‐value products. In particular, these diverse chemical features make lignin an attractive precursor for engineering functional materials such as porous carbons, where the aromatic backbone and reactive functional groups can be strategically leveraged to control carbon structure and surface chemistry.

### 2.3 Sources of Lignin

**FIGURE 3:**
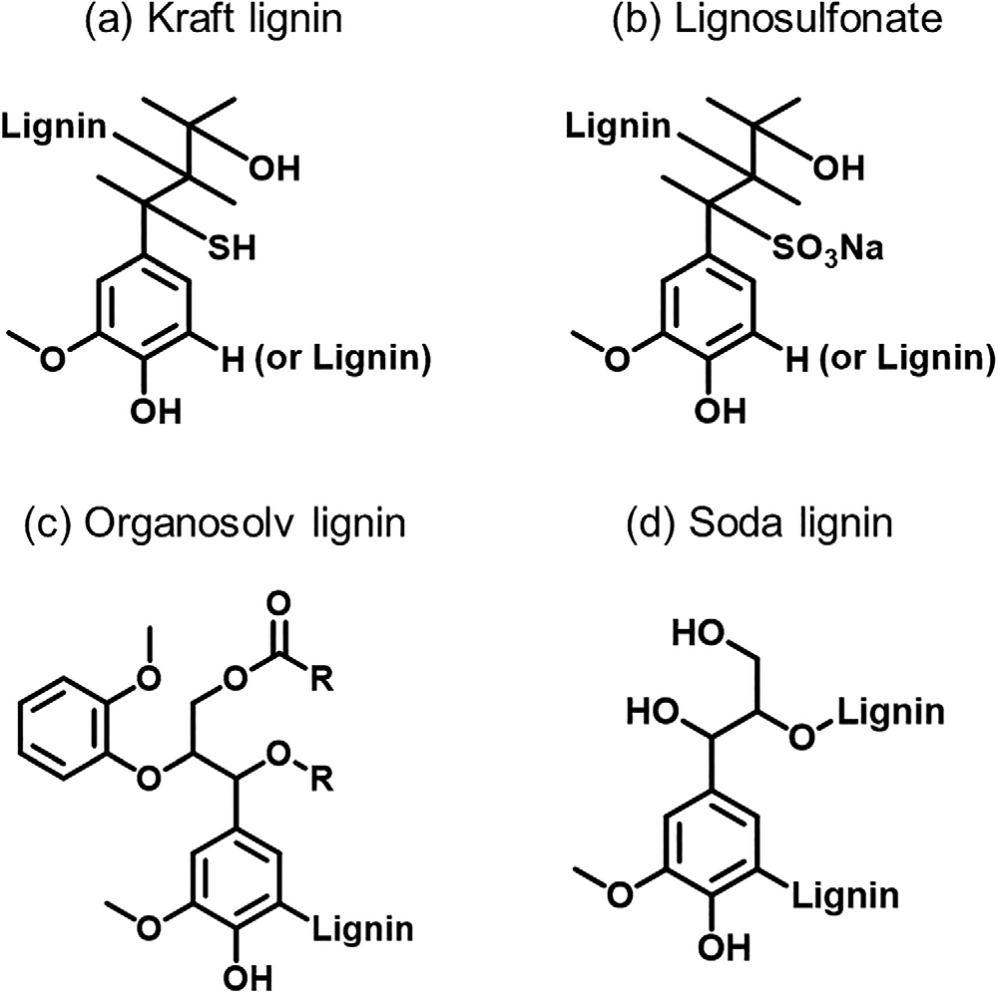
Simplified chemical structures of the four most common industrial lignin derivatives: (a) Kraft lignin (produced via the Kraft pulping process), (b) Lignosulfonate (derived from the sulfite pulping process), (c) Organosolv lignin (isolated using organic solvent extraction), and (d) Soda lignin (obtained through soda pulping).

Unlocking the full potential of lignin begins with its effective conversion into valuable products. A wide range of lignin can serve as feedstocks for such transformation processes. Currently, most industrial lignin, often referred to as technical lignin are generated as by‐products from delignification processes in the pulp and paper industry. These include kraft lignin, lignosulfonates, and soda lignin, all of which are produced at a large scale. A simplified chemical structure of various lignin is shown in Figure 3. In the future, even greater volumes of lignin are expected from emerging second‐generation biorefineries, which aim to valorize all components of lignocellulosic biomass (cellulose, hemicellulose, and lignin) in an integrated and sustainable manner. Despite the global pulp and paper industry producing tens of millions of tons of lignin annually, only a small fraction (less than 5%) is currently used in value‐added applications. The majority is burned on‐site as a low‐efficiency energy source. However, lignin obtained through alternative extraction methods, such as organosolv, soda, or ionic liquid‐based processes, offer higher purity and better reactivity, making them more suitable for advanced material applications and chemical synthesis. Table 1 provides a comparative overview of the main types of technical lignin commonly derived from industrial and emerging biomass pretreatment processes.

- ***Kraft lignin***: Kraft lignin, derived from the sulfate pulping process using sodium hydroxide and sodium sulfide, accounts for the majority of global lignin production. Although the Kraft process efficiently removes lignin from wood (up to 95%), it alters the native lignin structure, introducing sulfur‐containing groups and generating lignin rich in phenolic units and resistant C–C bonds. Additionally, Kraft lignin often contains residual carbohydrates and sulfur (1%–3%), which may limit its use in some high‐purity applications [47]. Nevertheless, its widespread availability and aromatic content make it an attractive candidate for conversion into functional materials, such as porous carbon for energy storage and gas adsorption.
- ***Lignosulfonate***: Lignosulfonate is water‐soluble lignin derived from the sulfite pulping process. They contain a variety of functional groups, including sulfonic, carboxylic, and phenolic groups, which contribute to their high molecular weight and complex structure. Due to their solubility and charge properties, lignosulfonates are commonly used as dispersing agents, surfactants, and binders. However, their high impurity content (up to 30 wt%) and incorporation of sulfonate groups often hinder catalytic valorization or material applications [48].
- ***Organosolv lignin***: Organosolv lignin is obtained by treating biomass with mixtures of organic solvents (e.g., ethanol, formic acid) and water under mild acidic conditions. This method produces high‐purity, sulfur‐free lignin with low molecular weight and excellent reactivity [49]. The process also cleanly separates cellulose, hemicellulose, and lignin into distinct streams, facilitating full biomass valorization. Organosolv lignin is highly suited for conversion into high‐value products such as fine chemicals, composites, and porous carbon materials, due to their clean structure and minimal contamination.
- ***Soda lignin***: Produced via soda or soda–anthraquinone pulping, soda lignin is mainly obtained from annual plants like straw, bagasse, and flax. This process avoids the use of sulfur, resulting in sulfur‐free lignin with relatively low molecular weights (1,000–3,000 Da) and fewer impurities. Although some ash contamination may occur due to residual sodium salts, soda lignin is often considered more chemically accessible than Kraft lignin and has shown potential in applications requiring clean lignin sources [49].
- ***Ionic liquid‐derived lignin***: Ionic liquids, low‐melting organic salts with negligible vapor pressure, have recently gained attention as green solvents for biomass fractionation. While still in early development, ionic liquid‐based methods can selectively extract lignin under mild conditions, yielding structurally diverse and relatively pure lignin fractions. This lignin show promise for future use in material synthesis and bio‐based chemical production, although their industrial scalability remains a challenge [50].

**TABLE 1:** Comparative overview of the main types of technical lignin commonly derived from industrial and emerging biomass pretreatment processes.

Type of lignin	Extraction process	Purity	Main features
Kraft lignin	Sulfate pulping (NaOH + Na_2_S, 165–175°C)	Moderate (impurities like carbs and sulfur)	Most widely available; rich in phenolics; contains recalcitrant C─C bonds and thiol groups
Soda lignin	Sulfur‐free alkaline pulping (NaOH, 140–170°C), often for annual crops	Relatively high	Sulfur‐free; low impurities; possible ash from soda
Lignosulfonate	Sulfite pulping (HSO_3_^−^ and SO_3_^2−^ ions)	Low (up to 30% impurities)	Water‐soluble; anionic polyelectrolytes; difficult to process; sulfonated aromatic structure
Organosolv lignin	Organic solvent pulping (e.g., ethanol + acid catalyst)	High	Sulfur‐free; clean separation; hydrophobic; good chemical reactivity
Ionic liquid‐derived lignin	Biomass solubilization using ionic liquids (e.g., imidazolium‐based salts)	Potentially very high	Emerging method; tunable; mild conditions; not yet industrialized

Overall, lignin represents not only a by‐product of the pulp and paper industry in need of better utilization, but also a valuable raw material that can be refined and upgraded in integrated biorefineries. The method used for lignin extraction significantly influences its molecular structure and reactivity, thereby affecting its suitability for various applications. Efficient pretreatment and separation strategies that yield consistent, high‐purity lignin is essential for the development of value‐added lignin‐based products. Among these, porous carbon materials derived from lignin are receiving increasing attention for use in CO_2_ capture, energy storage, and catalysis, reflecting a promising avenue for sustainable material design in line with green chemistry principles. It should be emphasized that the final pore structure and CO_2_ adsorption performance of lignin‐derived porous carbons are not determined by lignin type alone, but are predominantly governed by synthesis parameters, including the activation agent, templating strategy, carbonization temperature, and post‐treatment conditions. Different lignin types and extraction methods primarily influence the precursor properties, such as purity, ash content, molecular weight, and native heteroatom content, which can indirectly affect carbon yield, processability, and surface chemistry evolution during carbonization and activation. However, these feedstock‐related effects do not translate into a direct correlation between lignin type and adsorption capacity.

## 3 Preparation Techniques of Porous Carbon Materials Derived from Lignin

The preparation of lignin‐derived porous carbons has played a fundamental role in the development of high‐performance CO_2_ adsorbents. Conventional synthesis strategies, including chemical activation and templating methods, enable precise control over pore structure and surface chemistry, which are key factors governing CO_2_ capture performance. Chemical activation has considered as the most widely used approach due to its effectiveness in creating abundant microporosity and high surface area. In contrast, templating strategies allow for ordered pore architectures and hierarchical networks that facilitate mass transport. Despite differences in process complexity and environmental impact, these approaches provide fundamental insights into how processing parameters influence pore development, heteroatom incorporation, and ultimately CO_2_ capture performance. Establishing this foundation is critical not only for benchmarking advanced synthesis techniques but also for guiding the rational design of sustainable lignin‐derived carbons optimized for CO_2_ capture.

### 3.1 Chemical Activation Method

Activation is primarily intended to generate and enhance the porosity of carbon materials by forming internal voids and channels that promote gas diffusion and provide accessible adsorption sites. In chemical activation, these pores are developed through reactions between the carbon precursor and activating agents such as acids, bases, or salts under high‐temperature conditions. Depending on the synthesis route, chemical activation can be carried out via either a (1) conventional two‐step process or a (2) direct one‐step process.

#### 3.1.1 Conventional Two‐Step Process

**FIGURE 4:**
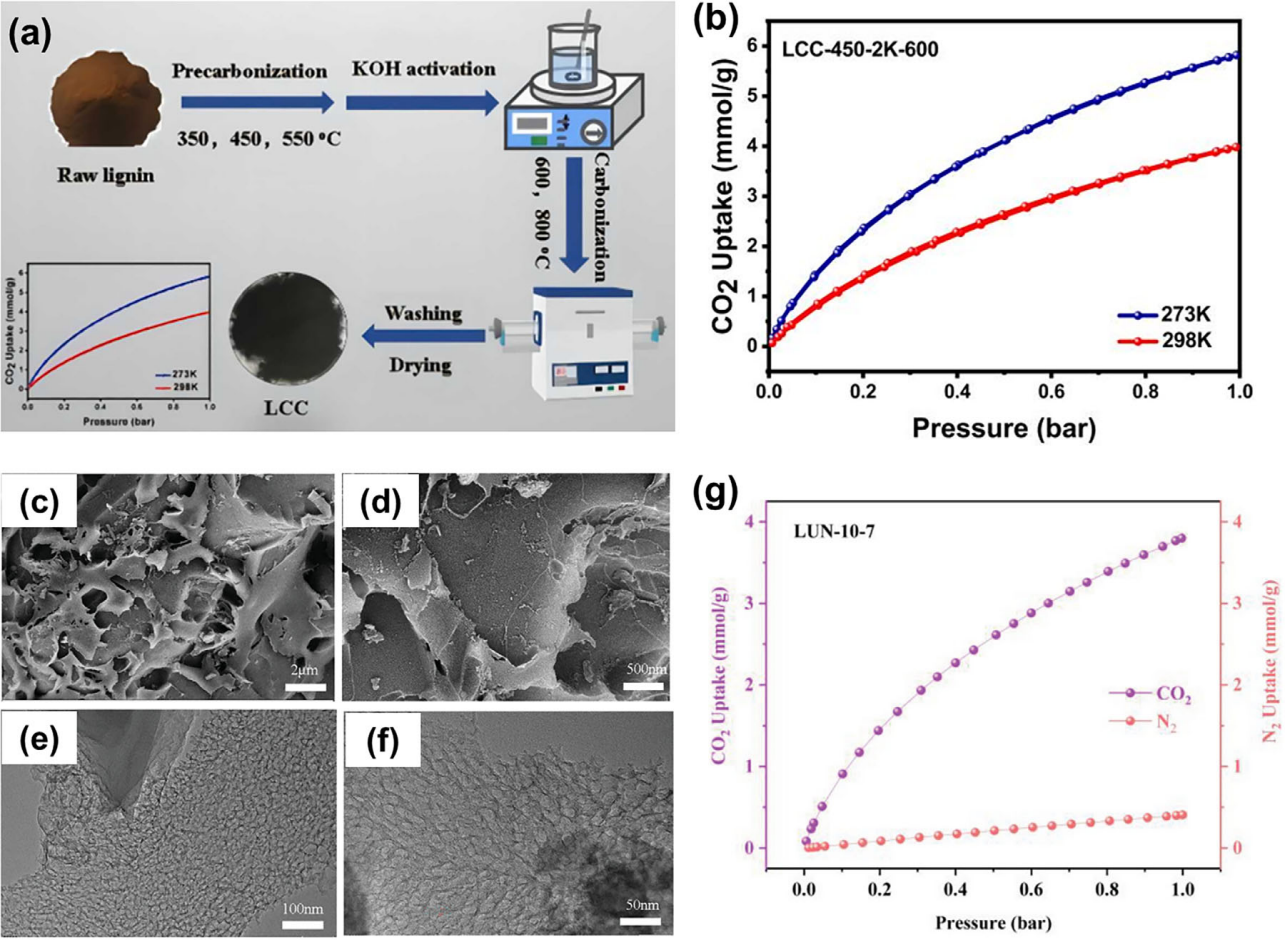
(a) Schematic diagram for the synthesis of lignin‐derived porous carbon, and (b) CO_2_ adsorption curves of porous carbon at different temperatures between 0 and 25°C. Reproduced with permission [51]. (c, d) SEM and (e, f) TEM images of N‐doped porous carbon (**LUN‐10‐7**), and (g) CO_2_/N_2_ selective adsorption of **LUN‐10‐7**. Reproduced with permission [38].

The conventional two‐step approach separates the degradation and porosity development steps. The first step covers the initial processing of the lignin precursor via drying and powdering, followed by high‐temperature carbonization to obtain carbon. The second step involves the impregnation of carbon with a chemical activating agent (e.g., KOH, NaOH, K_2_CO_3_, Na_2_CO_3_) in a solid‐state or liquid‐state fashion, and then subjected to further heat treatment at the desired temperature to generate and expand the pore network. As representative examples, recent studies exemplify how this method effectively tailors the porosity and surface chemistry of porous carbon derived from lignin for CO_2_ adsorption applications. Gong and Bao reported the preparation of N,O co‐doped porous carbon from lignin using a sequential pre‐carbonization and chemical activation strategy (Figure 4a) [51]. In their study, dealkalized lignin was first pre‐carbonized at selected temperatures (350–550°C) under nitrogen to achieve degradation and conversion into a carbonaceous intermediate, followed by KOH activation at various ratios and final carbonization at 600 or 800°C. The optimal material exhibited a well‐developed microporous structure with a specific surface area of 1493 m^2^/g and a micropore volume of 0.54 cm^3^/g. This material achieved CO_2_ adsorption capacities of 5.82 mmol/g at 273 K and 3.98 mmol/g at 298 K under 1 bar (Figure 4b), outperforming many previously reported biomass‐derived carbons. In a related study, the same group reported the fabrication of nitrogen‐doped porous carbon materials from lignin using sequential carbonization and post‐treatment activation/doping [38]. In this approach, lignin was first carbonized at 450°C to decompose its macromolecular structure, followed by impregnation in urea solution as a nitrogen source, and finally chemically activated using KOH at 600°C. Among the resulting N‐doped porous carbons, the optimized sample **LUN‐10‐7** was selected for detailed morphological characterization. The SEM images in Figure 4c,d reveal that **LUN‐10‐7** exhibits a flaky surface with fragmented debris visible at a magnification of 2 µm and irregular holes on the surface when further enlarged. The corresponding TEM images (Figure 4e,f) display numerous disordered worm‐like pores and folded surfaces, confirming the presence of a highly microporous architecture. These intertwined pores and pleated surfaces create abundant active sites, which are favorable for enhancing CO_2_ adsorption. The extensive microporosity is primarily generated by the aggressive chemical activation of lignin with KOH, which effectively develops the internal pore network and increases the accessible surface area. The optimized sample **LUN‐10‐7** achieved a CO_2_ adsorption capacity of 3.80 mmol/g at 298 K and 1 bar, along with good CO_2_/N_2_ selectivity (18.93) and strong cyclic stability over 10 adsorption‐desorption cycles (Figure 4g). The enhanced performance was attributed to the synergistic development of micropores (with a micropore surface area of 800 m^2^/g) and a relatively high nitrogen content (4.09 wt%), particularly in the form of pyrrole‐N and pyridine‐N functionalities that strengthened acid‐base interactions with CO_2_. While the overall CO_2_ uptake is moderate compared to some state‐of‐the‐art materials, this strategy highlights the tunability of nitrogen doping and pore structure through careful control of urea concentration and immersion time, making it a promising approach for scalable applications. The results of both studies demonstrate the effectiveness of the conventional two‐step process in precisely controlling porosity and surface chemistry, providing a simple yet environmentally friendly method for transforming lignin into high‐performance CO_2_ adsorbents.

In another example, Chen et al. developed N/S co‐doped porous carbon materials using lignin as the carbon precursor, KOH as the activator, and thiourea as a dual N and S source [52]. The synthesis involved pre‐carbonization of lignin, activation with KOH, and subsequent thiourea doping under N_2_ atmosphere. By optimizing the thiourea ratio and carbonization temperature, the authors obtained a sample (**NSPC‐700‐4**) that achieved a CO_2_ adsorption capacity of 3.49 mmol/g at 298 K and 1 bar. The superior performance was attributed to a balanced microporous structure combined with surface heteroatom functionalities. Specifically, S doping promoted the generation of N species (pyridine N and pyrrole N) and created oxidized S groups, both of which enhanced acid–base and polar interactions with CO_2_. The material also exhibited excellent CO_2_/N_2_ selectivity and cycling stability, highlighting the role of co‐doping in tuning both pore architecture and surface chemistry for effective CO_2_ capture. Building upon this, Feng et al. reported the preparation of N and S co‐doped ultramicroporous carbon materials using lignin as the carbon precursor and thiourea as the dopant under KOH activation [39]. The optimized sample exhibited a high specific surface area of 1435 m^2^/g, with ultramicropores (<1 nm) accounting for 67.8% of the pore volume, and achieved a CO_2_ adsorption capacity of 3.58 mmol/g at 298 K and 1 bar. Mechanistic analyses revealed that S doping facilitated the conversion of nitrogen into pyrrole N species, which provided abundant active sites and improved CO_2_ affinity. The combination of ultramicropore engineering and synergistic N/S functionalization was identified as the key factor for the superior adsorption performance. In addition, the adsorption process was characterized by a moderate heat of adsorption (∼18 kJ/mol), indicating predominantly physisorption with facile regenerability. These studies highlight a clear progression in lignin‐derived carbon research: from demonstrating the feasibility of N/S co‐doping to elucidating the mechanistic synergy between ultramicropores and heteroatom functionalities. Both works emphasize that precisely tuned ultramicropores (<1 nm) and heteroatom‐induced active sites are decisive in optimizing CO_2_ adsorption while maintaining regenerability, offering a sustainable and effective pathway for carbon capture.

**FIGURE 5:**
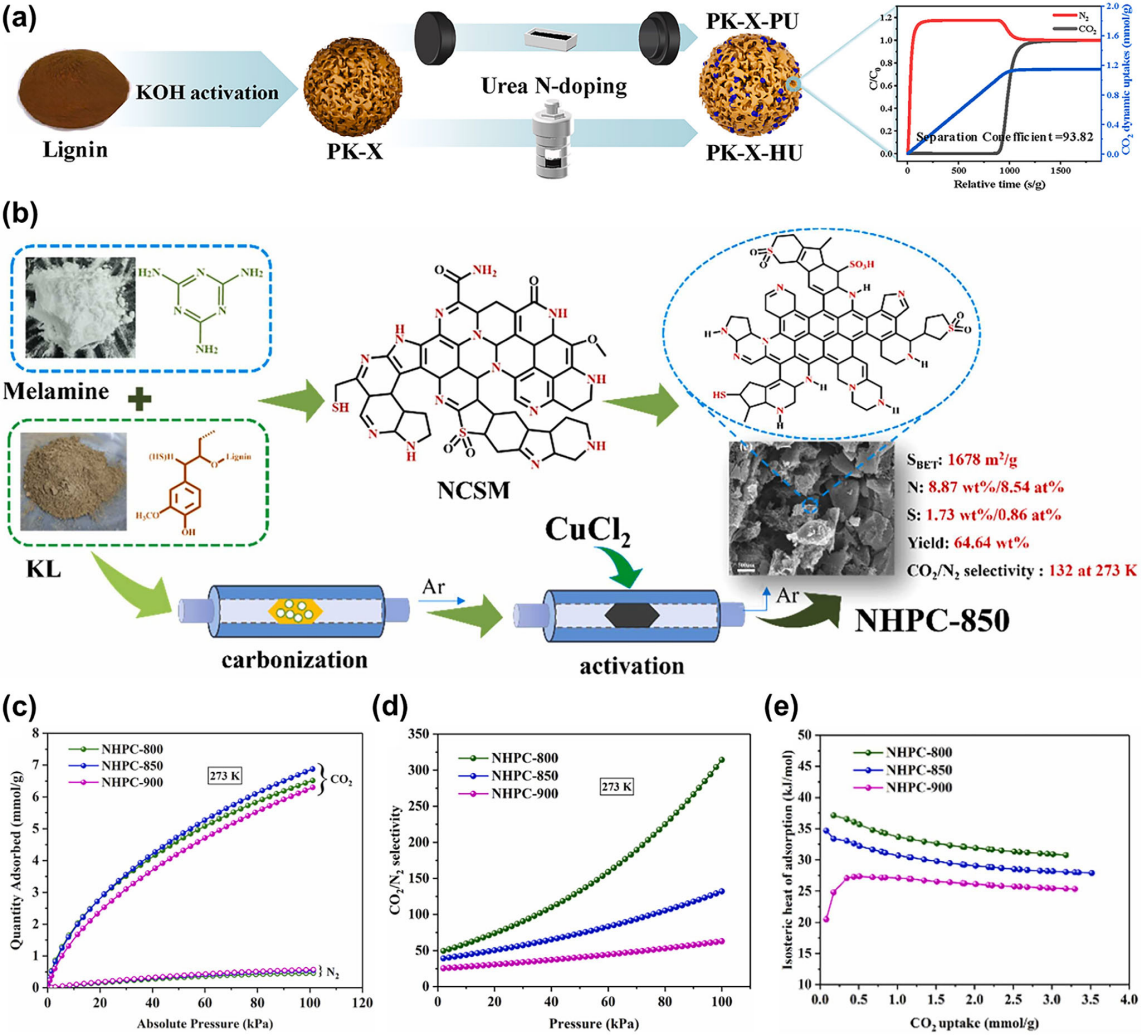
(a) Schematic diagram for the synthesis of N‐doped microporous carbon and CO_2_/N_2_ gas mixture penetration curve. Reproduced with permission [53]. (b) Schematic illustration of N, S co‐doped hierarchical porous carbon, (c) CO_2_ and N_2_ adsorption isotherms obtained at 273 K, (d) IAST CO_2_/N_2_ selectivity at 273 K, and (e) isosteric heat of CO_2_ adsorption for all the prepared materials. Reproduced with permission [27].

While the conventional two‐step method separates carbonization and activation, alternative approaches maintain simultaneous carbonization/activation treatments followed by a separate doping step. Dong et al. reported the preparation of lignin‐based porous carbon via thermochemical activation and post‐treatment nitrogen doping (Figure 5a) [53]. In this study, dealkalized lignin was first activated with KOH at 700°C to develop a porous structure, followed by nitrogen doping using urea through either pyrolysis or hydrothermal treatment. The optimized material exhibited a high specific surface area (1279 m^2^/g) with substantial ultramicroporosity and achieved an impressive CO_2_ adsorption capacity of 4.46 mmol/g at 25°C and 1 bar. Notably, the material also demonstrated excellent selectivity for CO_2_ over N_2_ (dynamic separation coefficient of 93.82) and superior cyclic stability. The authors highlighted that pore volume in the range of 0.6–0.8 nm primarily governed CO_2_ uptake under ambient conditions, while nitrogen functionalities (especially pyrrolic N) dominated adsorption performance at elevated temperatures or low CO_2_ partial pressures. This approach effectively decouples pore development and surface functionalization, enabling optimization of both properties independently. Bai et al. demonstrated the preparation of N, S co‐doped hierarchical porous carbon from lignin using a synergistic strategy of melamine modification and CuCl_2_ activation (Figure 5b) [27]. Unlike typical alkali activation, this method involved direct carbonization of lignin mixed with melamine at 500°C, followed by CuCl_2_ activation at 850°C, producing carbon with advanced hierarchical porosity and high heteroatom contents (8.54 at% N and 0.86 at% S). The optimized sample (**NHPC‐850**) exhibited a high specific surface area (1678 m^2^/g), a dominant narrow micropore volume (0.42 cm^3^/g), and achieved an excellent CO_2_ adsorption capacity of 6.87 mmol/g at 273 K and 1 bar, alongside exceptional CO_2_/N_2_ selectivity (132 at 273 K) (Figure 5c,d). Notably, this method provided a high material yield (64.64 wt%), highlighting its scalability and sustainability advantages over conventional KOH activation, which typically results in lower yields and heteroatom loss. The isosteric heat of CO_2_ adsorption (*Q*_st_) provides insight into the strength of interaction between CO_2_ molecules and the carbon surface. Based on the CO_2_ adsorption isotherms measured at 273 K and 303 K, the calculated *Q*_st_ for all N, S co‐doped porous carbon materials prepared at different carbonization temperatures range from 25 to 38 kJ/mol (Figure 5e), remaining below 40 kJ/mol. These values indicate a typical physical adsorption process, which is advantageous for the regeneration and reusability of the adsorbents. Among the samples, **NHPC‐850** exhibits a moderately high *Q*_st_ value of 34.7 kJ/mol at the initial CO_2_ loading, which consequently leads to strong interactions between the carbon surface and CO_2_ molecules. This enhanced interaction arises from the synergistic presence of N‐ and S‐containing functional groups together with abundant narrow micropores, which collectively increase the local electrostatic potential and facilitate CO_2_ binding. This result highlights the effectiveness of sequential carbonization/activation followed by heteroatom doping for tailoring porosity and surface chemistry, leading to materials with superior CO_2_ capture performance. While the adsorption capacities rival or exceed those from traditional two‐step KOH activation, the lower corrosivity and higher yield suggest this method holds strong promise for industrial applications.

#### 3.1.2 Direct One‐Step Process

In the direct one‐step process, carbonization and activation occur simultaneously. In this approach, the lignin precursor is first mixed with the desired activating agent. During carbonization, the pore structure is developed through two simultaneous reactions: (i) the degradation of lignin and the activating agent, and (ii) the chemical reactions between lignin and the activating species. To support the direct one‐step process, Li et al. reported the preparation of advanced bio‐carbon materials from Kraft lignin via a simple integrated carbonization‐activation method, where lignin was pre‐mixed with KOH and subjected to simultaneous carbonization and activation at 600–800°C [54]. The optimal material exhibited a high specific surface area of 1528 m^2^/g with a predominance of ultra‐microporosity (up to 93% of total pore volume), and achieved an outstanding CO_2_ adsorption capacity of 6.86 mmol/g at 0°C and 4.30 mmol/g at 25°C under 1 bar CO_2_. Notably, even at low partial pressure (15 kPa CO_2_), the material showed a remarkable uptake of 2.01 mmol/g at 25°C and excellent CO_2_/N_2_ selectivity (up to 38). The superior performance was attributed to the synergistic development of abundant ultramicropores (<0.5 nm) and potassium‐intercalated surface sites generated during the integrated process, which enhanced CO_2_ affinity via both pore‐filling and electrostatic interactions. This study clearly demonstrates the effectiveness of the direct one‐step method for producing lignin‐derived carbons with exceptional CO_2_ capture capability, while eliminating the complexity of post‐treatment steps. However, careful control of activation conditions is essential, as overly severe conditions may widen pores and reduce selectivity at low pressures.

**FIGURE 6:**
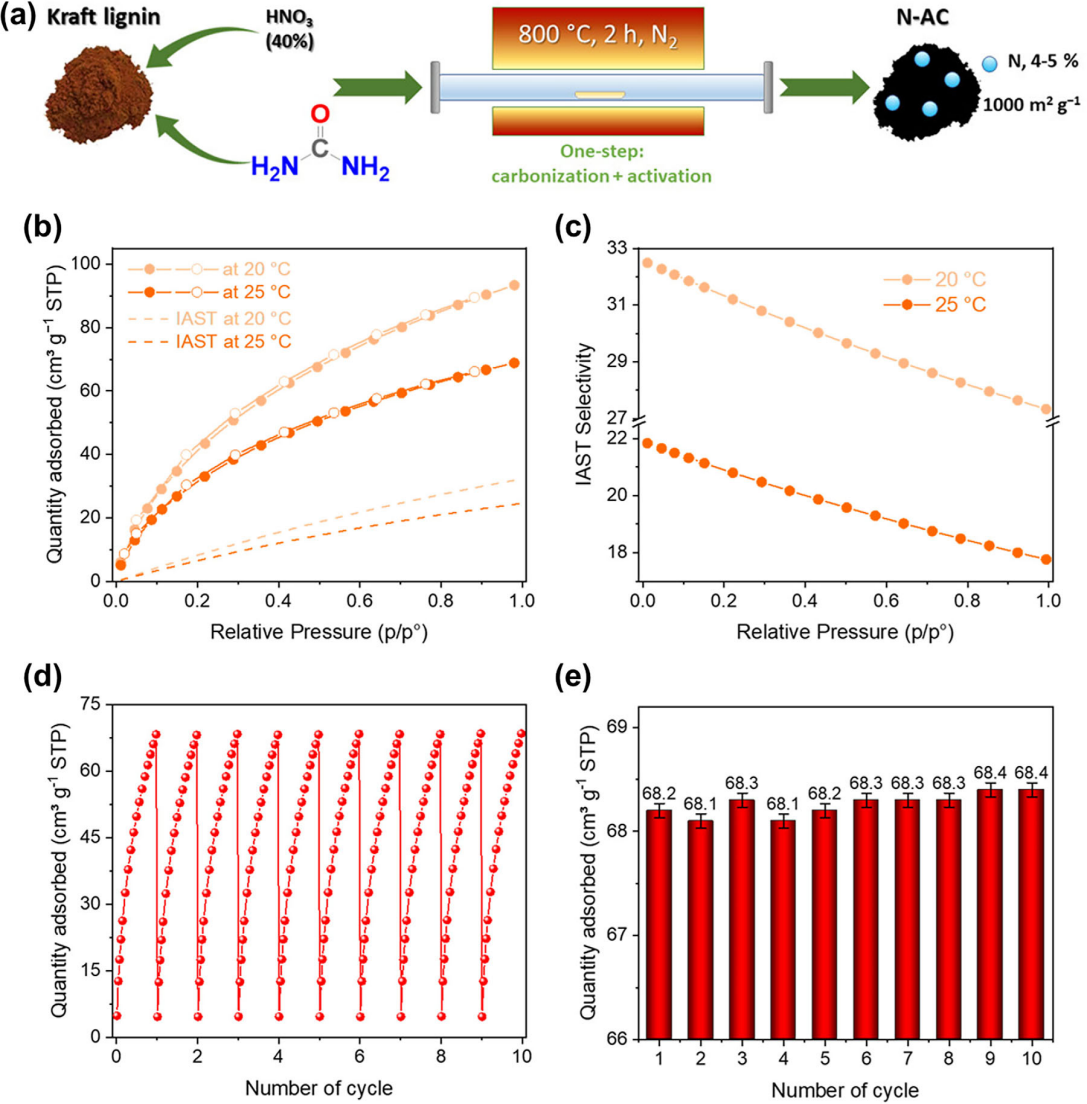
(a) Schematic diagram for the preparation of N‐doped activated carbon derived from Kraft lignin, (b) Adsorption (filled symbols) and desorption (hollow symbols) isotherms of CO_2_ at 20 and 25°C (solid lines) and simulated adsorption IAST isotherms of CO_2_ (dash lines) for gaseous mixture (15% of CO_2_ and 85% of N_2_), (c) CO_2_/N_2_ IAST selectivity of N‐doped activated carbon at 20°C and 25°C for artificial gaseous mixture consisting of CO_2_ (15%) and N_2_ (85%), (d) cyclic study of CO_2_ adsorption on N‐doped activated carbon at 25°C and at pressure within 0–0.98 bar, and (e) CO_2_ adsorption capacity of N‐doped activated carbon at 25°C and 1 bar during ten consequently adsorption–desorption cycles. Reproduced with permission [55].

Aside from utilizing the heteroatoms inherently present in lignin, heteroatom doping is a common strategy to tailor the surface chemistry of carbon materials derived from lignin for improved CO_2_ adsorption. Heteroatoms, such as nitrogen or sulfur, can enhance the affinity toward CO_2_ by introducing basic sites and polar functionalities. Moreover, the surface chemistry can also be adjusted depending on the type of technical lignin used, such as lignosulfonate or enzymatic hydrolysis lignin, which contain varying native amounts of nitrogen and sulfur, respectively. To support this discussion, recent studies have demonstrated that heteroatom doping significantly enhances CO_2_ adsorption performance in lignin‐derived carbons. Tkachenko et al. synthesized nitrogen‐doped activated carbon from Kraft lignin using a green, metal‐free one‐step process where nitric acid acted as the activator and urea as the N‐dopant (Figure 6a) [55]. The resulting carbon exhibited a high nitrogen content (4%–5%), a predominance of ultramicropores (<0.6 nm accounting for 86% of pore volume), and a specific surface area of 1000 m^2^/g. This material achieved a CO_2_ adsorption capacity of 4.2 mmol/g at 20°C and 3.1 mmol/g at 25°C under 1 bar (Figure 6b). The uptake decreased with temperature due to the exothermic nature of physical adsorption. The ideal adsorption solution theory (IAST) simulations (dashed lines) closely match the experimental data, indicating the reliability of the model in predicting gas separation behavior for a simulated flue gas mixture (15% CO_2_/85% N_2_). Under these conditions, the CO_2_ capacities were 1.4 mmol/g (20°C) and 1.1 mmol/g (25°C), confirming that the material maintains strong affinity toward CO_2_ even at low partial pressures representative of real flue gases. The calculated IAST selectivity curves show that this material exhibits excellent CO_2_/N_2_ selectivity, reaching 32.5 (0.1 bar) and 27.3 (1 bar) at 20°C. The values decrease as the temperature increases to 25°C (from 22.8 to 17.8), consistent with weakened physisorption at higher temperatures (Figure 6c). This strong selectivity results from the synergistic effect between nitrogen functionalities (pyridinic N and graphitic N) that enhance CO_2_–adsorbent electrostatic interactions and ultramicropores that provide size‐matching confinement for CO_2_ molecules. The recyclability of this material was evaluated through ten consequent adsorption–desorption cycles at 25°C. The presented isotherms (Figure 6d) and histograms (Figure 6e) show that N‐doped activated carbon keeps CO_2_ capture level without visible decay. In parallel, Saha et al. reported a one‐step synthesis of sulfur‐doped nanoporous carbons from lignin using Na_2_S_2_O_3_ as the sulfur source and KOH as the activating agent [56]. The best‐performing sample exhibited an exceptionally high specific surface area of 3626 m^2^/g, a total pore volume of 1.74 cm^3^/g, a sulfur content of ∼3.5 at%, and achieved a remarkable CO_2_ adsorption capacity of ∼11 mmol/g at 298 K and 1 bar. These results illustrate that both nitrogen and sulfur heteroatom doping, either derived from intrinsic lignin composition or introduced via additives, can be effectively utilized to tailor the surface chemistry and pore structure, significantly improving CO_2_ affinity. Importantly, the differences in sulfur or nitrogen content achievable also reflect the variability introduced by lignin type and its industrial processing history, emphasizing the tunability of such biomass‐derived carbons.

**FIGURE 7:**
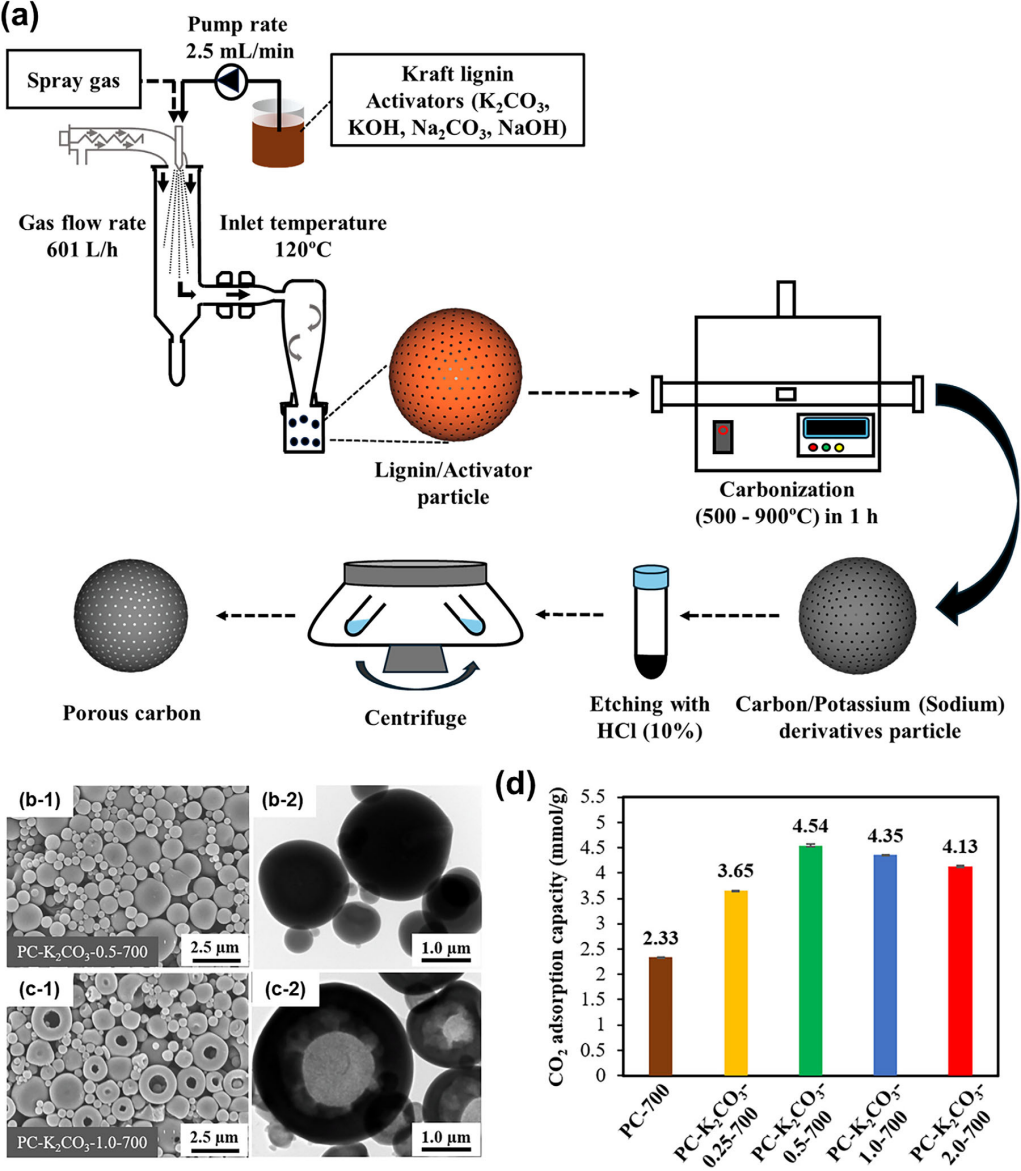
(a) Schematic diagram for the preparation of porous carbon particles derived from Kraft lignin and different activators, SEM and TEM images of carbon particles produced from Kraft lignin and K_2_CO_3_ at different K_2_CO_3_/lignin mass ratios of (b) 0.5 and (c) 1.0. (d) CO_2_ adsorption capacities of porous carbon materials synthesized at various K_2_CO_3_/lignin mass ratios. Reproduced with permission [28].

Among chemical activators, potassium carbonate (K_2_CO_3_) emerges as a particularly attractive green activator for producing lignin‐derived porous carbon. Compared to conventional KOH, K_2_CO_3_ offers significant advantages: it is less corrosive, low toxic, environmentally benign, and produces carbonate residues that are easier to handle, making it safer for long‐term equipment use and better suited for industrial‐scale applications. A recent study in our group demonstrated a sustainable synthesis approach that integrates K_2_CO_3_ activation with spray drying and subsequent carbonization, providing a scalable, efficient, and simple route for preparing porous carbon (Figure 7a) [28]. The spray drying technique ensured uniform mixing and precise stoichiometric control in a single‐step process, facilitating consistent particle morphology and homogeneous porosity [21, 57, 58, 59, 60]. This technique provides a versatile and relevant route for preparing a wide range of functional porous materials [61, 62, 63, 64, 65, 66]. As shown in SEM images, at a low K_2_CO_3_/lignin mass ratio of 0.5, the carbon particles exhibit uniform morphologies with spherical structures (Figure 7b‐1). However, as the K_2_CO_3_/lignin mass ratio increases to 1.0, some particles become more irregular morphologies with numerous cavities on the surface (Figure 7c‐1). Notably, by adjusting the K_2_CO_3_/lignin mass ratio, the internal structure of carbon particles could be easily controlled from a compact to a hollow structure, as demonstrated in TEM images (Figure 7b‐2, c‐2). The optimized material (**PC‐K_2_CO_3_‐0.5‐700**), prepared at 700°C with a K_2_CO_3_/lignin mass ratio of 0.5, exhibited a high specific surface area (1308.8 m^2^/g), abundant ultramicroporosity (0.554 cm^3^/g), and delivered an impressive CO_2_ adsorption capacity of 4.54 mmol/g at 298 K and 1 bar (Figure 7d). This performance was comparable to those activated with KOH and outperforming materials activated with NaOH or Na_2_CO_3_ under identical conditions. Building on this progress, Wang et al. reported the preparation of lignin‐waste–derived porous carbon via a one‐step direct activation/carbonization strategy using K_2_CO_3_ [67]. Optimization of the activator concentration and carbonization temperature yielded the best material (**L‐K‐2–750**), synthesized at 750°C with a lignin/K_2_CO_3_ ratio of 3:2. This sample exhibited a specific surface area of 797 m^2^/g, total pore volume of 0.33 cm^3^/g, and achieved a CO_2_ adsorption capacity of 3.75 mmol/g at 25°C and 1 bar (4.47 mmol/g at 0 °C and 1 bar). Importantly, it showed excellent CO_2_/N_2_ selectivity (57.2 at 15:85 CO_2_/N_2_), low regeneration energy with isosteric heats of 15.9–23.8 kJ/mol, and stable cycling over five adsorption–desorption cycles with <2% capacity loss. These studies establish K_2_CO_3_ as an environmentally friendly activator for producing sustainable lignin‐derived porous carbon. They also highlight the decisive role of ultramicroporosity and optimized activation conditions in maximizing CO_2_ uptake, while demonstrating excellent selectivity, stability, and regenerability essential for practical carbon capture applications.

#### 3.1.3 Other Multistep Processes

**FIGURE 8:**
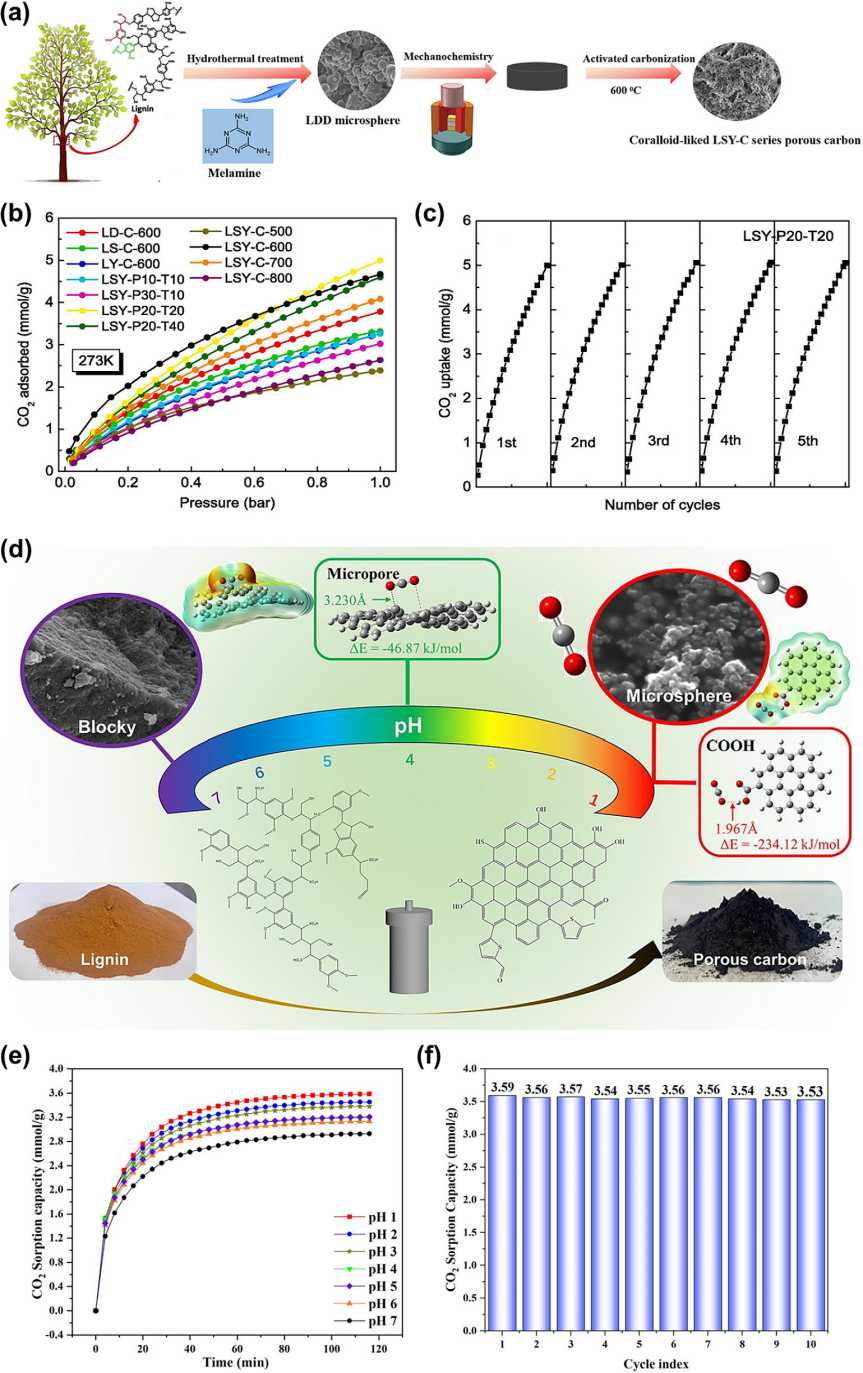
(a) Schematic diagram for the fabrication of N‐doped porous carbon particles derived from enzymatic hydrolysis lignin, (b) CO_2_ adsorption isotherms (at 273 K) of N‐doped porous carbon particles synthesized at different preparation conditions, and (c) cycle performance of the optimized sample (**LSY‐P20‐T20**) at 273 K and 1 bar. Reproduced with permission [68]. (d) Schematic diagram for the preparation of porous carbon materials derived from sodium lignosulfonate, (e) CO_2_ adsorption capacity (at 25°C) of porous carbon materials synthesized at different acid treatments (from pH 1 to pH 7), and (f) cycle performance of the optimized sample (modified by acid at pH 1) at 25°C and 1 bar. Reproduced with permission [69].

Multistep processes have been employed to introduce heteroatoms or modify the pore structure of carbon materials. In some cases, additional heat treatments are performed beyond the conventional two‐step process of carbonization and activation, such as physical activation to further enhance specific surface area or post‐treatment doping to enrich surface functionalities that promote CO_2_ adsorption. Although these extra processing steps can significantly improve adsorbent performance, they also lead to greater process complexity and higher production costs. For instance, Liu et al. reported a sophisticated method for preparing nitrogen‐doped porous carbon from enzymatic hydrolysis lignin [68]. Their strategy combined hydrothermal treatment, mechanochemical pressing, and KOH activation at low temperatures (Figure 8a). This integrated process resulted in carbons with tunable porosity, high microporosity (∼70%), and ultrahigh surface oxygen contents (30.93%–55.32%). The optimized sample (**LSY‐P20‐T20**) achieved a specific surface area of 1233 m^2^/g, a narrow micropore volume (d < 1.0 nm) of 0.27 cm^3^/g, and exhibited an impressive CO_2_ adsorption capacity of 5.00 mmol/g at 273 K and 1 bar (Figure 8b). The enhanced performance was attributed to the synergy between abundant ultramicropores and surface nitrogen and oxygen functional groups, which improved both CO_2_ adsorption capacity and CO_2_/N_2_ selectivity. Furthermore, **LSY‐P20‐T20** was tested for cyclic stability through five consecutive CO_2_ adsorption–desorption cycles at 273 K and 1.0 bar, and almost no decline in capacity was found (Figure 8c), demonstrating that N‐doped porous carbon had good cycling application ability for CO_2_ capture. As another example, Gao et al. employed a hydrothermal acid pretreatment prior to carbonization and CO_2_ physical activation to tailor the morphology and adsorption performance of lignin‐based porous carbons (LPCs) derived from sodium lignosulfonate (Figure 8d) [69]. In this approach, acid pretreatment significantly altered carbon morphology, transforming it from blocky particles to uniform microspheres with higher degrees of graphitization and enriched carboxyl groups. The optimized sample, which was modified by acid at pH 1, exhibited a specific surface area of 1018 m^2^/g and delivered the CO_2_ adsorption capacity of 3.67 mmol/g at 25°C and 1 bar (Figure 8e), while maintaining excellent stability over ten adsorption‐desorption cycles (Figure 8f). Importantly, density functional theory (DFT) calculations confirmed that carboxyl groups contributed the strongest electrostatic attraction to CO_2_ molecules, explaining the enhanced adsorption performance at lower temperatures where surface functionalities dominate. These studies highlight the effectiveness of a hydrothermal pretreatment step to adjust microporosity, mesoporosity, and surface chemistry synergistically, leading to excellent CO_2_ capture performance. While such multi‐step routes introduce greater complexity and processing time compared to simpler two‐step routes, it enables fine control over material properties and illustrate a pathway toward the rational design of lignin‐derived adsorbents tailored for practical CO_2_ capture applications.

### 3.2 Templating Methods

In the field of pore structure engineering, ordered porous materials are a class of materials that exhibit long‐range order and size homogeneity in their pore structure [70, 71]. Templating approaches are widely employed to create such ordered porosity [72, 73]. The structure of the final material is directed by the use of a sacrificial template with a predefined and controlled morphology, which is later removed to reveal the desired porous framework [74, 75, 76, 77, 78, 79, 80, 81].

**FIGURE 9:**
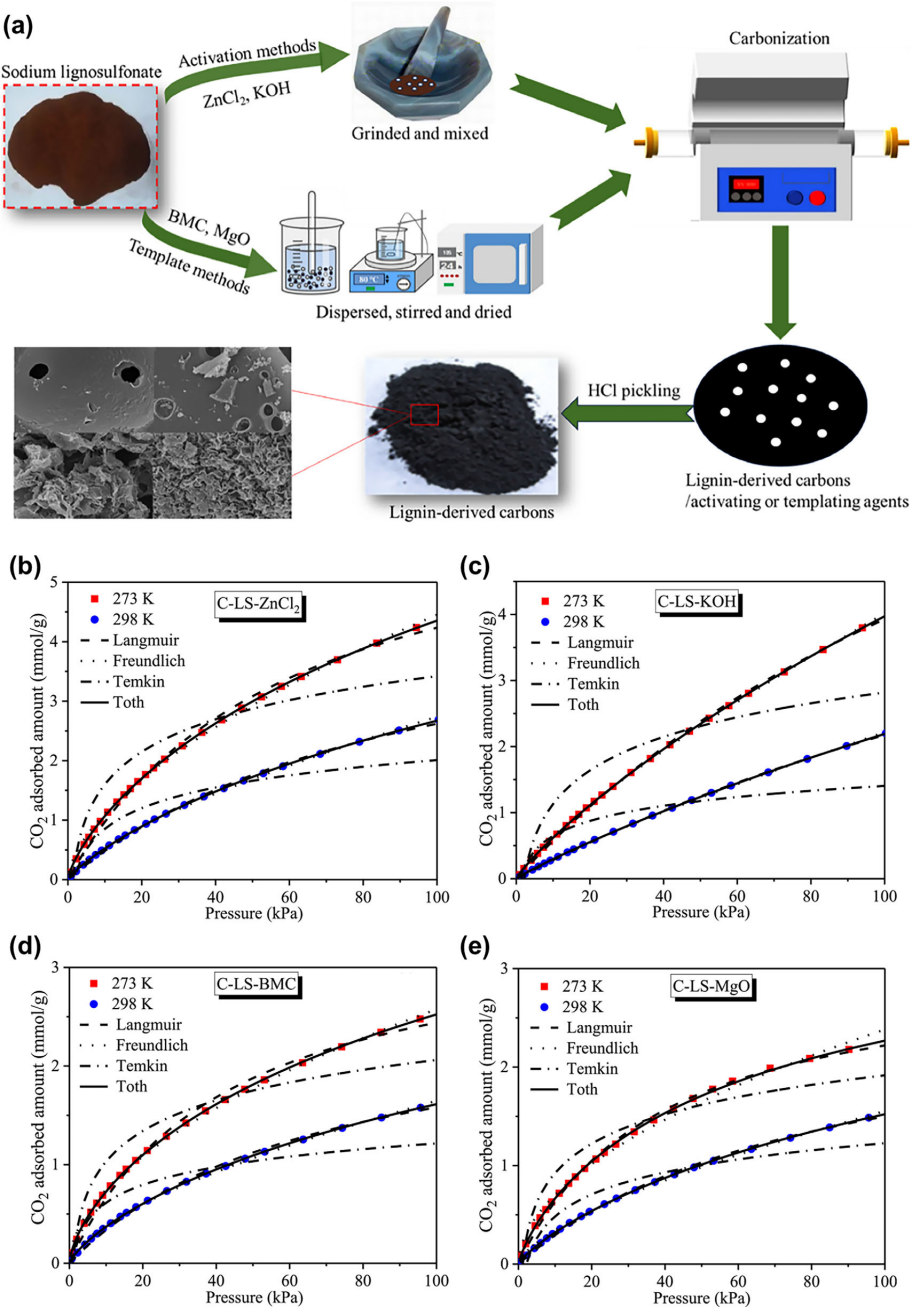
(a) Schematic illustration for the preparation of porous carbon derived from sodium lignosulfonate using activation method and template method, CO_2_ adsorption isotherms at 273 K and 298 K along with nonlinear isotherm model fittings for porous carbon derived from sodium lignosulfonate using (b) ZnCl_2_ and (c) KOH as activation agents, and (d) BMC and (e) MgO as templating agents. Reproduced with permission [83].

To support the application of templating methods in preparing lignin‐derived porous carbons for CO_2_ capture, two related studies by Zhao and co‐workers provide valuable insights. In an earlier report, they used black liquor lignin as a precursor and compared chemical activation (KOH and ZnCl_2_) with templating approaches using basic magnesium carbonate (BMC) and MgO [82]. The template‐derived carbons (**C‐BLL‐BMC** and **C‐BLL‐MgO**) showed distinct morphologies, including slit‐like mesopores and hollow structures, but lower surface areas (681 and 362 m^2^/g) compared to the chemically activated samples. CO_2_ adsorption capacity reached up to 2.20 mmol/g at 0°C for **C‐BLL‐BMC** and 2.01 mmol/g for **C‐BLL‐MgO**, indicating that while templating imparted ordered mesoporosity, it yielded lower microporosity and adsorption capacity compared to KOH‐activated carbon (5.20 mmol/g at 0°C). In a subsequent publication, the same group extended this approach to sodium lignosulfonate as the carbon source, again comparing template methods (BMC and MgO) with chemical activation (KOH and ZnCl_2_) (Figure 9a) [83]. The templated carbons derived from sodium lignosulfonate exhibited layered structures with mesopore‐rich textures and moderate specific surface areas (738 and 1020 m^2^/g), whereas chemically activated carbons demonstrated much higher specific surface areas (1998 m^2^/g for KOH and 1125 m^2^/g for ZnCl_2_) and larger micropore volumes. The CO_2_ adsorption isotherms at 273 K and 298 K for these samples are shown in Figure 9b–e. Among all samples, the ZnCl_2_‐activated carbon (**C‐LS‐ZnCl_2_**) achieved a CO_2_ adsorption capacity of 4.45 mmol/g at 273 K and 1 bar, outperforming the template‐derived carbons, which achieved significantly lower capacities. The CO_2_ adsorption capacity decreased with increasing temperature to 298 K, reflecting the exothermic nature of CO_2_ physisorption. To gain deeper insight into the CO_2_ adsorption equilibrium behavior of these porous carbon samples, the experimental adsorption isotherms were fitted using the Langmuir, Freundlich, Temkin, and Toth models. Among these models, the Toth model provided the best correlation (*R* ^2^ > 0.999), indicating that the adsorption occurred on a heterogeneous surface with varied adsorption energies. These two studies illustrate a clear trend: while templating methods produce well‐defined and ordered pore structures, they tend to result in lower microporosity and adsorption performance compared to chemical activation techniques, which better promote micropore formation essential for CO_2_ capture at ambient conditions. Notably, the later study demonstrated that ZnCl_2_ activation can yield high CO_2_ uptake with better control of pore structure and surface chemistry when applied to sodium lignosulfonate, a different lignin source. This comparison highlights the ability of the templating method to control pore morphology but also demonstrates its limitations in maximizing adsorption capacity. These findings suggest that combining templating with chemical activation might be a promising direction for future studies aiming to balance pore structure precision and adsorption performance.

**FIGURE 10:**
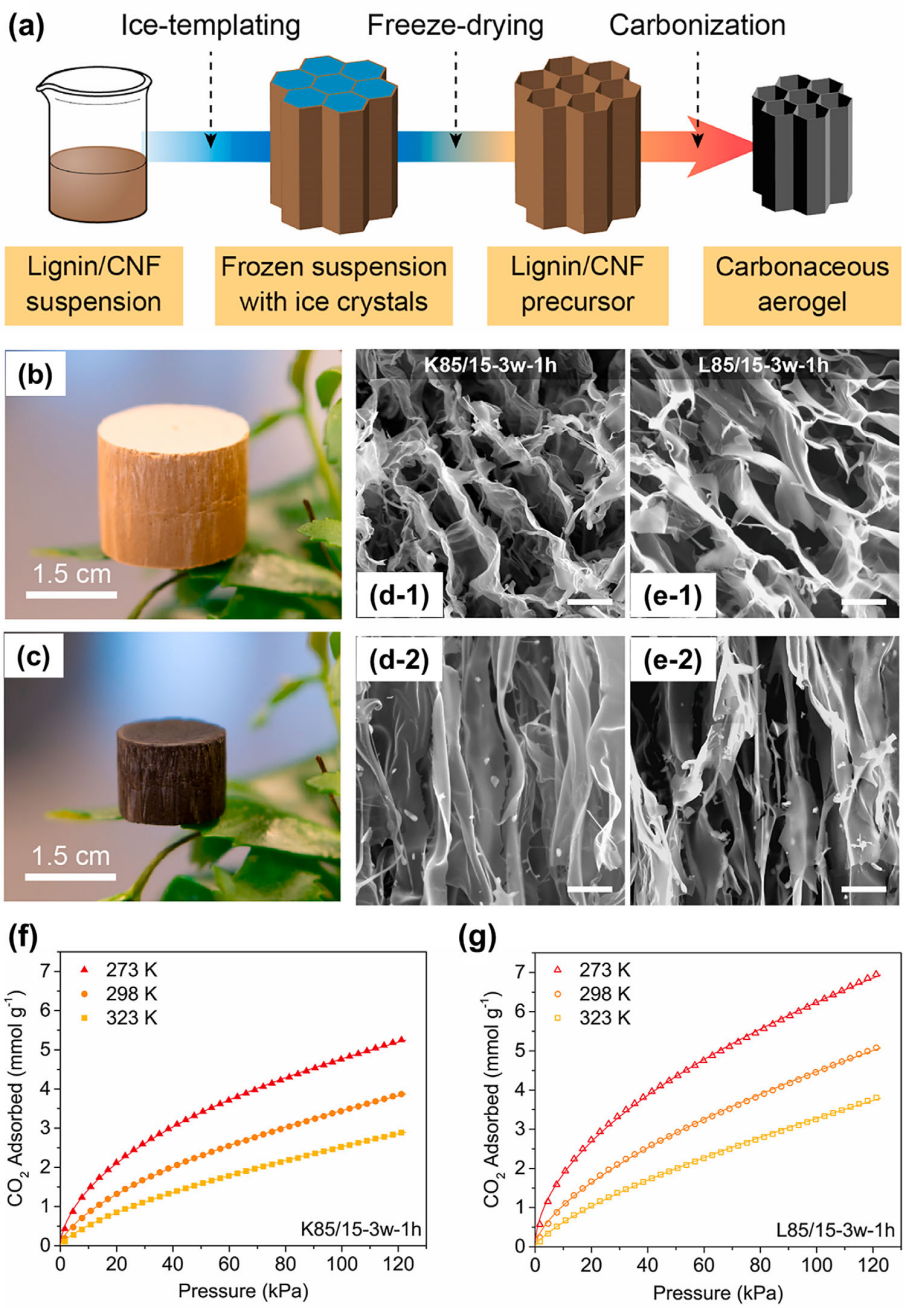
(a) Schematic preparation processing from lignin/CNF aqueous suspensions to carbon aerogels, and representative illustrations of (b) lignin/CNF precursor and (c) carbon aerogel. SEM images of carbon aerogels derived from (d) Kraft lignin and (e) Lignoboost lignin. CO_2_ adsorption isotherms at different temperatures of carbon aerogels derived from (f) Kraft lignin and (g) Lignoboost lignin. Reproduced with permission [85].

Given their unique hierarchical pore structure, low density, and excellent mechanical properties, carbon aerogels derived from lignin have attracted attention as effective materials for CO_2_ adsorption, as demonstrated in two studies by Geng and co‐workers. In an earlier report, they developed a strategy combining Kraft lignin and TEMPO‐oxidized cellulose nanofibers (TOCNFs) to fabricate hierarchical carbon aerogels via ice‐templating followed by carbonization at 1000°C [84]. The TOCNFs served as both structural support and sacrificial templates, enabling the formation of anisotropic hierarchical porous structures with tunable pore size distribution. By varying the TOCNF content, they achieved specific surface area up to 806 m^2^/g and CO_2_ adsorption capacity reaching 5.23 mmol/g at 273 K and 1 bar after post‐synthesis washing, which significantly improved performance by removing pore‐blocking sodium salts. The hierarchical architecture, containing interconnected macropores, mesopores, and micropores, facilitated rapid mass transfer and high adsorption efficiency. Building on this concept, their next study refined the process by optimizing material composition (lignin type, lignin/TOCNF ratio), suspension solid content, and carbonization time, while maintaining ice‐templating as the structuring approach [85]. The synthesis process, from the preparation of lignin/CNF suspensions to the formation of carbon aerogels, is shown in Figure 10a. Representative images of the lignin/CNF precursor and carbon aerogel are presented in Figure 10b,c, respectively. As revealed by the SEM images (Figure 10d,e), both carbon aerogels derived from Kraft lignin and Lignoboost lignin exhibit anisotropic structures with well‐aligned longitudinal macropores due to the ice‐templating technique. The CO_2_ adsorption isotherms of both carbon aerogels at different temperatures are evaluated and the results are shown in Figure 10f,g. The optimized carbon aerogel derived from Lignoboost lignin, which is a purer form of lignin with lower ash content and higher phenolic hydroxyl groups, demonstrated an improved CO_2_ adsorption capacity of 6.28 mmol/g at 273 K and 1 bar. This value surpasses the performance of the material reported in their earlier study [84]. Moreover, this monolithic carbon aerogel exhibited excellent CO_2_/N_2_ selectivity (∼21), hydrophobicity (low water vapor uptake), and robust mechanical integrity, enabling binder‐free packing for dynamic CO_2_ capture applications. These studies illustrate a clear advancement: the later work improved not only adsorption capacity and selectivity but also mechanical robustness and scalability by employing Lignoboost lignin and fine‐tuning processing parameters. This progression exemplifies how careful selection of lignin source and optimization of the templating process can significantly enhance both the structural and functional performance of lignin‐derived porous carbons. These results highlight the potential of ice‐templating as a scalable and sustainable approach for producing monolithic adsorbents with tailored porosity and high CO_2_ capture efficiency.

Sani and co‐workers investigated the combination of lignin‐derived mesoporous carbons with amine functionalization to create high‐performance CO_2_ adsorbents [86]. They synthesized three‐dimensional mesoporous carbon materials from Kraft lignin using mesocellular foam (MCF) silica as a template via a solvothermal process, followed by carbonization and template removal. By changing the template‐to‐lignin ratio and carbonization temperature, and comparing MCF‐templated carbons to those templated with commercial two‐dimensional mesoporous silica, they demonstrated that the MCF‐derived carbons, featuring a 3D interconnected pore network with large mesopore volumes (up to 1.80 cm^3^/g), allowed higher polyethylenimine (PEI) loading and more uniform amine dispersion. As a result, the best‐performing sample (MC2‐800‐1 impregnated with 60 wt% PEI) achieved a CO_2_ uptake of 2.90–3.13 mmol/g at 75–90°C and 0.15 bar under 15% CO_2_/N_2_, which was significantly higher than analogous PEI sorbents prepared using 2D silica templates. These MCF‐derived sorbents also exhibited faster adsorption kinetics, superior amine efficiency (up to 0.30 mol CO_2_/mol‐N), and excellent cyclic stability over 50 adsorption–desorption cycles. This study highlights that the large pore size and 3D connectivity of MCF‐templated carbons are critical for maximizing PEI utilization and ensuring rapid CO_2_ diffusion, offering a promising strategy to design efficient and regenerable solid sorbents for post‐combustion capture.

In summary, the synthesis strategy strongly influences the textural and adsorption properties of lignin‐derived porous carbons. Switching between microporous and mesoporous regimes generally requires a change in synthesis strategy rather than a change in lignin type, because the dominant pore‐forming mechanism is imposed by the chosen preparation route. Chemical activation predominantly generates micropores and ultramicropores through chemical etching, intercalation, and gasification reactions, making it the most effective approach for developing abundant ultramicropores (<0.7 nm), which are critical for high CO_2_ uptake under low‐pressure conditions. The choice of activator and processing conditions directly determines pore development and surface chemistry, with greener agents such as K_2_CO_3_ offering a promising alternative to conventional alkali activators (e.g., KOH, NaOH). Direct one‐step activation further simplifies processing while maintaining high selectivity and regenerability. In contrast, templating methods intrinsically impose mesoporous or macroporous frameworks defined by the geometry of the template, and changing the template size primarily modifies mesopore dimensions rather than converting them into micropores. As a result, templating methods generate ordered mesoporous or macroporous networks that facilitate rapid gas diffusion and post‐functionalization but generally produce lower microporosity, resulting in moderate CO_2_ adsorption capacities. While lignin type and extraction method influence precursor properties such as ash content, heteroatom availability, and reactivity, they do not fundamentally alter the pore‐forming mechanism governed by the synthesis route. Hybrid or multi‐step approaches, integrating hydrothermal or mechanochemical pretreatments with activation, enable fine control over surface functionalities (e.g., N/O/S species) and hierarchical porosity, thereby balancing high adsorption capacity with stability and reusability. These comparisons highlight that optimizing ultramicroporosity (<0.7 nm) and surface chemistry is the key to maximizing CO_2_ adsorption efficiency, while excessive mesoporosity mainly improves mass transfer kinetics rather than equilibrium capacity. To provide a clearer overview of these distinct preparation routes, the main characteristics of the chemical activation and templating methods are summarized in Table 2. The table compares their processing principles, pore‐forming mechanisms, dominant pore types, and corresponding effects on CO_2_ adsorption performance. This comparative summary highlights how each approach governs pore architecture and surface chemistry, thereby dictating the adsorption capacity, selectivity, and practical applicability of lignin‐derived porous carbons for CO_2_ capture.

**TABLE 2:** Comparison of chemical activation and templating methods for preparing lignin‐derived porous carbon materials derived from lignin for CO_2_ capture.

Aspect	Chemical activation method	Templating method
Typical strategy/reagents	One‐ or two‐step activation using chemical agents such as KOH, NaOH, K_2_CO_3_, Na_2_CO_3_, ZnCl_2_; sometimes combined with heteroatom dopants (N, S, O) or hydrothermal pretreatments	Formation of carbon framework around sacrificial templates (hard templates: MgO, BMC, SiO_2_, NaCl; or soft templates: surfactants, block copolymers, ice‐templating). The template is removed after carbonization
Reaction conditions	Carbonization and activation generally occur at 600–900°C under N_2_; the activator ratio and temperature govern pore evolution	Carbonization at 700–1000°C; template removal by acid leaching or dissolution; often requires an additional washing step
Main pore‐forming mechanism	Chemical reactions between the carbon matrix and the activator (etching, gasification, intercalation) create micropores and ultramicropores (<0.7 nm)	Template geometry dictates ordered mesoporous or macroporous structures; pore size reflects template particle or assembly dimensions
Dominant pore type	Microporous/ultramicroporous, sometimes hierarchical when activation is controlled	Mesoporous or hierarchical, usually with lower micropore content
Surface chemistry tunability	Easy to introduce heteroatoms (N, S, O) via dopants or functional precursors; enhances CO_2_ affinity through acid–base and polar interactions	Limited heteroatom incorporation unless combined with separate doping or post‐functionalization steps
Typical CO_2_ adsorption capacity	3–11 mmol/g at 1 bar, 0–25°C; dominated by ultramicropore filling and heteroatom sites	2–6 mmol/g at 1 bar, 0–25°C; governed by mesopore‐assisted diffusion; lower equilibrium capacity
Selectivity and regeneration	High CO_2_/N_2_ selectivity (up to 130); physisorption with moderate *Q*_st_ ≈ 20–38 kJ/mol ensures low energy regeneration	Moderate selectivity (∼15–25); diffusion‐enhanced kinetics
Advantages	Simple process; high specific surface area; excellent control of microporosity; tunable surface chemistry	Produces ordered and hierarchical pores; facilitates amine impregnation for chemisorption
Disadvantages	Corrosive activators (especially KOH, NaOH) and waste treatment issues; over‐activation may collapse pores	Multi‐step and time‐consuming; template removal generates waste and may cause structural damage
Representative CO_2_ adsorption trend	Capacity increases with ultramicropore volume (<0.7 nm) and heteroatom content	Capacity improves with larger mesopore volume when combined with post‐functionalization (e.g., PEI impregnation)

Table 3 provides a comparative summary of lignin‐derived porous carbons reported for CO_2_ capture, highlighting the relationships between processing method, textural properties, and CO_2_ adsorption capacity. From a comparative and practical perspective, these results indicate that the optimal synthesis strategy is application‐dependent rather than universally defined. Specifically, chemical activation is identified as the most effective approach for maximizing equilibrium CO_2_ adsorption capacity due to its ability to generate abundant ultramicropores (<0.7 nm), which are critical for pore‐filling‐driven CO_2_ capture under low‐pressure conditions and are supported by extensive experimental validation. In contrast, templating methods are more suitable for applications requiring enhanced mass‐transfer kinetics, hierarchical porosity, or post‐functionalization (e.g., amine impregnation), despite their generally lower equilibrium adsorption capacities. Hybrid and multi‐step approaches offer a balanced performance by integrating high adsorption capacity with improved diffusion and stability; however, they remain limited by increased process complexity and current challenges in large‐scale implementation.

**TABLE 3:** Summary of lignin‐derived porous carbons for CO_2_ capture, showing preparation method, textural properties, and CO_2_ adsorption capacity under different conditions.

	Material	Feedstock	Process	SSA	Pore size	CO_2_ adsorption capacity	References
1	N,O‐codoped porous carbon	Dealkalized lignin	Pyrolysis Chemical activation (KOH) and carbonization	1493 m^2^/g	Micropores (90.9%), Few mesopores	3.98 mmol/g at 25°C (1 bar) 5.82 mmol/g at 0°C (1 bar)	[51]
2	N‐doped porous carbon	Lignin (Aladdin)	Pyrolysis Chemical activation (KOH) and carbonization	1110 m^2^/g	Micropores (0.9‐1.7 nm, 67.8%), Few mesopores (2.4‐4.2 nm)	3.80 mmol/g at 25°C (1 bar)	[38]
3	N,S‐codoped porous carbon	Lignin (Aladdin)	Pyrolysis Chemical activation (KOH) and carbonization	1353 m^2^/g	Micropores (0.71‐1.69 nm, 89.3%), Few mesopores	3.49 mmol/g at 25°C (1 bar)	[52]
4	N,S‐codoped porous carbon	Lignin (Aladdin)	Pyrolysis Chemical activation (KOH) and carbonization	1435 m^2^/g	Ultramicropore (<1 nm, 67.8%), Few mesopores	3.58 mmol/g at 25°C (1 bar) 4.55 mmol/g at 0°C (1 bar)	[39]
5	N‐doped porous carbon	Organosolv lignin (Lignol Innovation Company)	Pyrolysis Chemical activation (KOH) and carbonization	1788 m^2^/g	Micropores (0.84‐2 nm, 53.8%), Few mesopores (2‐5 nm)	4.8 mmol/g at 25°C (1 bar) 8.2 mmol/g at 0°C (1 bar)	[87]
6	Porous carbon	Lignin protobind 2400 (ALM Private Limited, India)	Pyrolysis Chemical activation (KOH) and carbonization	1108 m^2^/g	Micropores (91.6%), Few mesopores	3.66 mmol/g at 25°C (1 bar) 5.68 mmol/g at 0°C (1 bar)	[88]
7	Porous carbon	Lignin	Pyrolysis Chemical activation (KOH) and carbonization	2224 m^2^/g	Micropores (0.6‐1.2 nm, 89.2%), Few mesopores	4.5 mmol/g at 25°C (1 bar) 7.3 mmol/g at 0°C (1 bar)	[89]
8	Porous carbon	De‐alkaline lignin (TCI America)	Pyrolysis Chemical activation (KOH) and carbonization	2254 m^2^/g	Micropores (0.45‐0.82 nm, 89.5%), Few mesopores	7.24 mmol/g at 25°C (1 bar) 9.4 mmol/g at 0°C (1 bar)	[90]
9	N‐doped porous carbon	Dealkalized lignin (TCI Shanghai)	Chemical activation (KOH) and carbonization Pyrolysis or hydrothermal carbonization	1279 m^2^/g	Ultramicropore (<1 nm, 61.4%), Mesopores (>10 nm)	2.97 mmol/g at 50°C (1 bar) 4.46 mmol/g at 25°C (1 bar)	[53]
10	N,S‐codoped porous carbon	Lignin (Rizhao Huatai Paper Industry)	Carbonization Chemical activation (CuCl_2_) and carbonization	1678 m^2^/g	Ultramicropore (<1 nm, 52.5%), Mesopores (5‐40 nm, 23.8%)	3.57 mmol/g at 30°C (1 bar) 6.87 mmol/g at 0°C (1 bar)	[27]
11	Porous carbon	Kraft lignin (Sigma‐Aldrich)	Chemical activation (KOH) and carbonization	878 m^2^/g	Ultramicropore (<0.7 nm, 74.7%), Few mesopores	4.34 mmol/g at 25°C (1 bar) 5.84 mmol/g at 0°C (1 bar)	[54]
12	N‐doped porous carbon	Softwood Kraft lignin (UPM BioPiva, Finland)	Chemical activation (HNO_3_) and carbonization	1000 m^2^/g	Micropore (0.7–1.1 nm, 82.6%), Few mesopores	1.2 mmol/g at 25°C (0.15 bar) 3.4 mmol/g at 25°C (1 bar)	[55]
13	S‐doped porous carbon	Dealkaline lignin (TCI America)	Chemical activation (KOH) and carbonization	3626 m^2^/g	Micropores (0.47–1.93 nm, 82.7%), Mesopores (2.0–4.5 nm, 17.3%)	10.89 mmol/g at 25°C (1 bar)	[56]
14	Porous carbon	Cross‐linked Kraft lignin (Kuraray Co., Ltd., Japan)	Chemical activation (K_2_CO_3_) and carbonization	1308.8 m^2^/g	Ultramicropore (<0.7 nm, 95.8%), Few mesopores	4.54 mmol/g at 25°C (1 bar)	[28]
15	Porous carbon	Lignin (Guangzhou Institute of Energy Conversion, Chinese Academy of Sciences)	Chemical activation (K_2_CO_3_) and carbonization	797 m^2^/g	Micropores (<2 nm, 90.9%), Few mesopores (2–4 nm)	3.75 mmol/g at 25°C (1 bar) 4.47 mmol/g at 0°C (1 bar)	[67]
16	N‐doped microporous carbon	De‐alkaline lignin (TCI America)	Chemical activation (KOH) and carbonization	2779 m^2^/g	Micropores (0.47–1.9 nm, 79.1%), Mesopores (20.9%)	5.48 mmol/g at 25°C (1 bar) 8.56 mmol/g at 0°C (1 bar)	[91]
17	N‐Mg‐functionalized porous carbon	Lignin (M Pulp and Paper Manufacturing Company, South Korea)	Mg impregnation (MgCl_2_) and carbonization	818 m^2^/g	Micropores (<2 nm), Few mesopores	3.02 mmol/g at 25°C (1 bar)	[92]
18	Porous carbon	Enzymatic hydrolysis lignin (China Oil & Foodstuffs Corporation)	Chemical activation (KOH) and physical activation (humidified N_2_) in microwave	2870 m^2^/g	Micropores (0.5–2.0 nm, 34.7%), Mesopores (2.0–8.0 nm, 65.3%)	1.31 mmol/g at 30°C	[29]
19	Porous carbon	Alkali lignin (Solar bio, Beijing)	Pyrolysis at negative pressure (−0.1 MPa)	1577.5 m^2^/g	Micropores (<2 nm, 48.7%), Mesopores (2.0–4.0 nm, 67.6%)	3.62 mmol/g at 0°C	[93]
20	N‐doped porous carbon	Enzymatic hydrolysis lignin	Hydrothermal treatment Mechanochemical treatment Chemical activation (KOH) and carbonization	1233.2 m^2^/g	Micropores (0.6–2.0 nm, 50%), Mesopores (2–20 nm)	2.7 mmol/g at 25°C (1 bar) 5.0 mmol/g at 0°C (1 bar)	[68]
21	Porous carbon	Sodium lignosulfonate (Shanghai Aladdin Biochemical Technology)	Hydrothermal treatment Physical activation (CO_2_) and carbonization	1018.1 m^2^/g	Mainly macropores and mesopores, Few micropores	3.67 mmol/g at 25°C (1 bar) 5.10 mmol/g at 0°C (1 bar)	[69]
22	N‐doped porous carbon	Lignosulfonate acid sodium salt	Hydrothermal treatment Chemical activation (KOH) and carbonization Post‐doping process	3021 m^2^/g	Micropores (<2 nm), Few mesopores (2‐5 nm)	2.6 mmol/g at 25°C (1 bar) 13.6 mmol/g at 25°C (10 bar)	[94]
23	PEI‐functionalized porous carbon	Lignin (Lignol Energy Corporation, Burnaby, BC, Canada)	Hydrothermal treatment Chemical activation (KOH) and carbonization Amine impregnation	1341 m^2^/g	Micropores (<2 nm), Mesopores (2‐8 nm)	1.5 mmol/g at 30°C (without PEI impregnation) 2 mmol/g at 30°C (with 5 wt% PEI impregnation)	[95]
24	Porous carbon	Black liquor lignin (alkali pulping company, Hunan, China)	Chemical activation (ZnCl_2_) and carbonization	896.4 m^2^/g	Micropores (<2 nm, 89.1%), Few mesopores	2.26 mmol/g at 25°C (1 bar) 2.96 mmol/g at 0°C (1 bar)	[82]
			Chemical activation (KOH) and carbonization	1336.5 m^2^/g	Micropores (<2 nm, 93.2%), Few mesopores	3.60 mmol/g at 25°C (1 bar) 5.20 mmol/g at 0°C (1 bar)	
			Template (Basic magnesium carbonate) and carbonization	681.0 m^2^/g	Micropores (<2 nm, 58.7%), Mesopores	1.75 mmol/g at 25°C (1 bar) 2.20 mmol/g at 0°C (1 bar)	
			Template (MgO) and carbonization	361.6 m^2^/g	Micropores (<2 nm, 44.6%), Mesopores	1.43 mmol/g at 25°C (1 bar) 2.01 mmol/g at 0°C (1 bar)	
25	Porous carbon	Sodium lignosulfonate (Shanghai Yien Chemical Technology Co., Ltd.)	Chemical activation (ZnCl_2_) and carbonization	1125.0 m^2^/g	Micropores (<2 nm, 65.5%), Mesopores (34.5%)	2.68 mmol/g at 25°C (1 bar) 4.45 mmol/g at 0°C (1 bar)	[83]
			Chemical activation (KOH) and carbonization	1998.4 m^2^/g	Micropores (<2 nm, 76.5%), Mesopores (23.5%)	2.20 mmol/g at 25°C (1 bar) 3.98 mmol/g at 0°C (1 bar)	
			Template (Basic magnesium carbonate) and carbonization	738.1 m^2^/g	Micropores (<2 nm, 20%), Mesopores (80%)	1.62 mmol/g at 25°C (1 bar) 2.53 mmol/g at 0°C (1 bar)	
			Template (MgO) and carbonization	1020.0 m^2^/g	Micropores (<2 nm, 27%), Mesopores (73%)	1.52 mmol/g at 25°C (1 bar) 2.24 mmol/g at 0°C (1 bar)	
26	N,S‐codoped porous carbon	Sodium lignosulfonate (Aladdin Technology Co., Ltd.)	Template (NaCl) and carbonization	824 m^2^/g	Micropores (0.5‐2 nm, 63.4%), Mesopores (2‐5 nm)	2.2 mmol/g at 25°C (1 bar) 3.4 mmol/g at 0°C (1 bar)	[96]
	S‐doped porous carbon			1078 m^2^/g	Micropores (0.5‐2 nm, 33.3%), Mesopores (2‐5 nm)	2.4 mmol/g at 25°C (1 bar) 3.8 mmol/g at 0°C (1 bar)	
27	Carbon aerogel	Kraft lignin (Sigma‐Aldrich, Sweden AB)	Ice‐templating Thermal stabilization Carbonization	1101 m^2^/g	Micropores (<2 nm), Mesopores (2‐4 nm)	5.23 mmol/g at 0°C (1 bar)	[84]
28	Carbon aerogel	Lignoboost lignin (Domtar Plymouth pulp mill, USA)	Ice‐templating Thermal stabilization Carbonization	380 m^2^/g	Micropore (<2 nm)	4.49 mmol/g at 25°C (1 bar) 6.28 mmol/g at 0°C (1 bar)	[85]
29	PEI‐functionalized mesoporous carbon	Lignin (Sigma‐Aldrich)	Template (mesocellular foam silica) and carbonization	900 m^2^/g	Mesopores (10–100 nm)	2.95 mmol/g at 75°C (0.15 bar) 3.13 mmol/g at 85°C (0.15 bar)	[86]

## 4 Textural and Surface Properties of Lignin‐Derived Porous Carbon for CO_2_ Capture

The efficiency of CO_2_ adsorption in lignin‐derived porous carbon materials is predominantly determined by their pore structure, including specific surface area, pore size distribution, pore volume, and pore architecture (micropores, mesopores, and macropores). These structural parameters significantly influence the adsorption capacity, selectivity, and kinetics of CO_2_ capture, particularly under post‐combustion conditions.

### 4.1 Importance of Micropores and Ultramicropores

**FIGURE 11:**
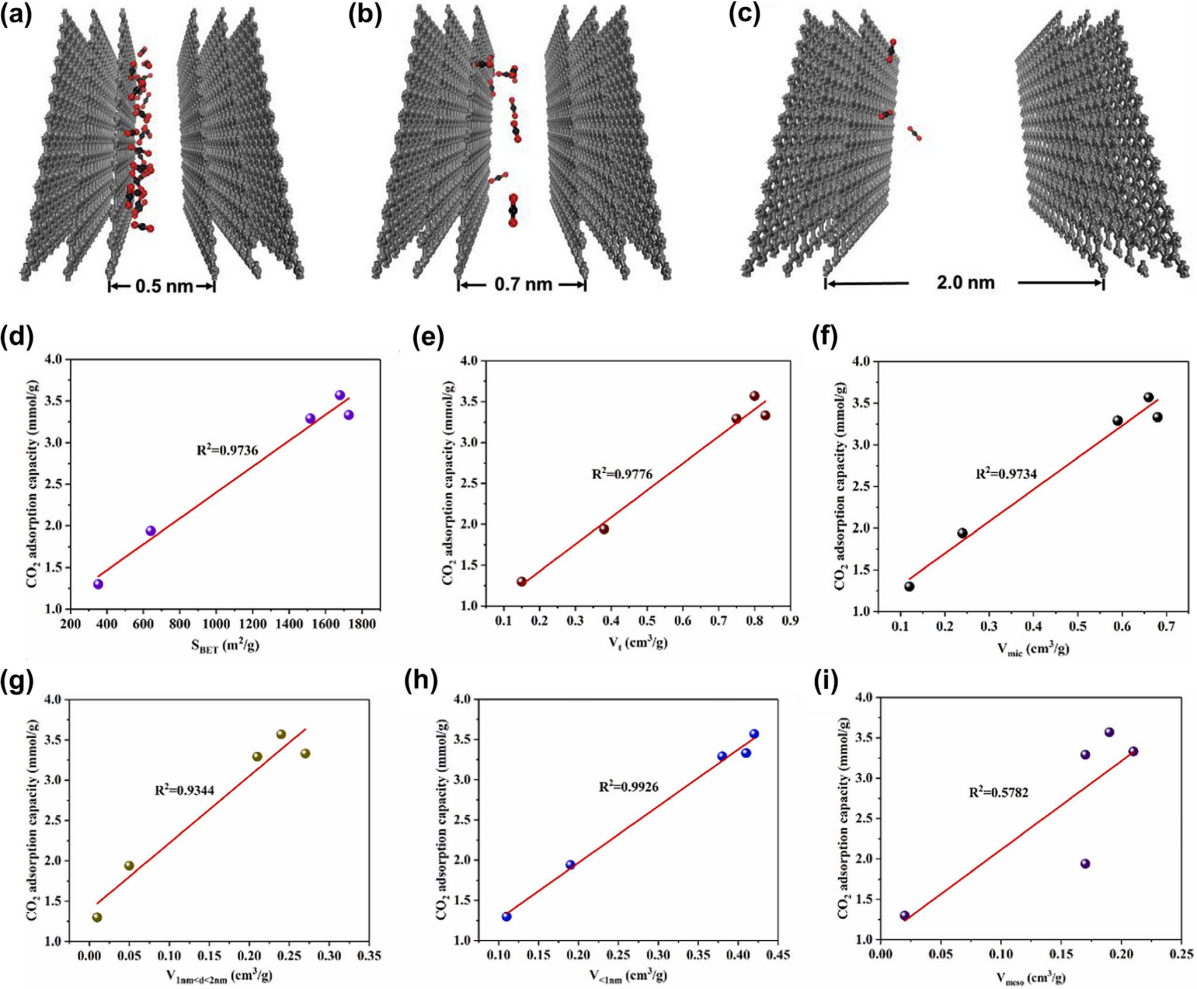
Comparisons for movies of CO_2_ loading in the perfect graphite slit pores with pore width of (a) 0.5 nm, (b) 0.7 nm, and (c) 2.0 nm derived from molecular simulations based on GCMC. Reproduced with permission [97]. Correlation between CO_2_ adsorption capacity and (d) specific surface area, (e) total pore volume, (f) micropore volume, (g) micropore volume of 1–2 nm, (h) narrow micropore (<1 nm), and (i) mesopore volume. Reproduced with permission [27].

The CO_2_ adsorption capacity of lignin‐derived porous carbons is primarily determined by their microporous framework, particularly the presence of ultramicropores with widths below 0.7 nm. Because the kinetic diameter of CO_2_ molecules (∼0.33 nm) closely matches these pore dimensions, confinement within ultramicropores generates strong van der Waals and quadrupole interactions, resulting in high adsorption potentials. This explains why ultramicropores predominantly contribute to CO_2_ uptake under post‐combustion conditions, where the CO_2_ partial pressure is relatively low (∼0.15 bar). This phenomenon is further supported by molecular simulations (GCMC) of CO_2_ loading in graphite slit pores with different pore widths (Figure 11a–c) [97]. The simulated density distributions reveal that CO_2_ molecules are densely confined when the pore width is approximately 0.5 nm, indicating nearly complete pore filling due to strong solid–gas interactions. In contrast, the adsorption density decreases as the pore size increases to 0.7 nm and becomes sparse at 2.0 nm, where the interaction between CO_2_ and the pore walls weakens due to diminished overlap of potential fields. These results demonstrate that strong confinement within ultramicropores (∼0.5 nm) substantially enhances adsorption potential, enabling efficient CO_2_ packing even at low pressures. The simulation findings are consistent with experimental observations showing that carbons with a high fraction of ultramicropores exhibit the highest CO_2_ uptake, confirming that optimal pore widths below 0.7 nm provide the strongest adsorbent–adsorbate interactions and govern CO_2_ adsorption capacity.

Numerous studies have demonstrated that micropores (<2 nm) provide the majority of accessible adsorption sites in lignin‐derived carbons [27, 28, 51, 54]. Nitrogen adsorption/desorption isotherms of these materials typically exhibit a sharp increase at very low relative pressures (*P*/*P*_0_ < 0.01), which is characteristic of microporous structures. For instance, Gong and Bao reported that N,O‐codoped porous carbon derived from lignin using KOH as an activator exhibited micropore fractions exceeding 90% of the total pore volume, with specific surface areas as high as 2604 m^2^/g and micropore volumes up to 0.96 cm^3^/g [51]. The optimized sample (**LCC‐350–2K‐600**), with a micropore surface area of 1965 m^2^/g and a *V*_micro_/*V*_total_ ratio above 90%, achieved CO_2_ uptakes of 5.82 mmol/g at 273 K and 3.98 mmol/g at 298 K. Similarly, Li et al. demonstrated that Kraft lignin‐derived carbons prepared through an integrated carbonization–activation process exhibited exceptionally high ultramicroporosity, with up to 92% of the micropores having pore widths below 0.7 nm [54]. The optimal sample (**KLK‐1–600**) achieved CO_2_ uptakes of 3.29 mmol/g at 0°C and 2.01 mmol/g at 25°C under 15 kPa CO_2_, confirming that well‐developed ultramicroporous structures are the most critical factor for enhancing CO_2_ capture at low partial pressures. These studies confirm that a high proportion of micropores, particularly ultramicropores, is essential for maximizing CO_2_ adsorption capacity, as they provide strong confinement and enhanced interaction potentials for CO_2_ molecules. The strong influence of pore structure on CO_2_ adsorption capacity was further verified by correlation analyses reported by Bai et al. (Figure 11d–i) [27]. A clear linear relationship (*R* ^2^ > 0.90) was observed between CO_2_ uptake and the specific surface area, total pore volume, and micropore volume, highlighting the critical role of microtextural parameters in adsorption. Among these, the correlation was most pronounced for narrow micropores with widths below 1 nm (*R* ^2^ = 0.9926), confirming that ultramicropores provide the most effective adsorption sites due to the optimal confinement of CO_2_ molecules. In contrast, the correlation weakened for wider micropores (1–2 nm) and became negligible for mesopores (*R* ^2^ = 0.5782), indicating that mesopores mainly facilitate gas diffusion rather than direct adsorption. These findings clearly demonstrate that ultramicropore enrichment, rather than total surface area, plays the dominant role in determining CO_2_ adsorption performance in lignin‐derived porous carbons.

While the total micropore volume correlates with CO_2_ uptake at higher pressures, under flue‐gas‐like conditions, the abundance of ultramicropores (<0.7 nm) is the most decisive factor. Their narrow pore widths significantly enhance the interaction energy between CO_2_ molecules and the carbon walls, resulting in relatively high isosteric heats of adsorption (*Q*_st_) for lignin‐derived carbons. For instance, Liu et al. reported that enzymatic‐hydrolysis‐lignin‐derived carbons exhibited CO_2_ uptakes up to 5 mmol/g at 273 K, with adsorption capacity determined primarily by narrow micropores (<1.0 nm). The corresponding *Q*_st_ values ranged from 23.4 to 46.3 kJ/mol, confirming that the process is dominated by physisorption [68]. Similarly, Bai et al. demonstrated that N,S co‐doped carbons displayed *Q*_st_ values between 25 and 38 kJ/mol, reinforcing that ultramicropore confinement provides the optimal balance between strong CO_2_ binding and efficient regenerability [27]. These results highlight that effective CO_2_ capture in lignin‐derived carbons arises from the synergy of abundant ultramicropores and favorable adsorption energetics: strong binding at low coverage to ensure selectivity, yet sufficiently moderate interaction energies to allow energy‐efficient regeneration. These features are crucial for practical post‐combustion capture applications.

The synthesis route plays a decisive role in the formation of ultramicropores. KOH activation is widely recognized as an effective method, generating micropores through etching and intercalation reactions; however, excessive activation ratios or very high carbonization temperatures (>700–800°C) can cause micropores to widen into mesopores or even lead to partial collapse of the pore walls. Gong and Bao observed that reducing the carbonization temperature from 800 to 600°C promoted denser ultramicroporosity and improved CO_2_ affinity, whereas higher temperatures led to pore merging and reduced uptake [51]. Similarly, pretreatment steps such as pre‐carbonization can degrade lignin's cross‐linked structure and create more uniform activation sites, thereby promoting micropore development without the need for harsh chemical treatments. Recent studies using K_2_CO_3_ as a more environmentally friendly activator for Kraft lignin have demonstrated similar behavior [28]. When the carbonization temperature was increased from 500 to 700°C, the CO_2_ adsorption capacity improved markedly from 2.61 to 4.54 mmol/g due to the growth of ultramicropore volume (from 0.308 to 0.554 cm^3^/g). However, further heating to 900°C reduced both the ultramicropore volume (to 0.506 cm^3^/g) and CO_2_ uptake (3.98 mmol/g), indicating that excessive carbonization temperature results in pore widening and diminished confinement. Similarly, the activator‐to‐lignin mass ratio also governs pore development. At a low K_2_CO_3_/lignin mass ratio of 0.5, ultramicropore volume and CO_2_ adsorption capacity (4.54 mmol/g at 298 K) were maximized, whereas increasing the K_2_CO_3_/lignin mass ratio to 2.0 generated abundant mesopores (*V*_meso_ = 1.837 cm^3^/g) but decreased CO_2_ adsorption capacity (4.16 mmol/g), since mesopores do not provide effective confinement at low pressures. These findings clearly demonstrate that both carbonization temperature and activator concentration must be carefully optimized to preserve ultramicroporosity and prevent structural degradation, thereby ensuring high‐performance CO_2_ capture.

Although ultramicropores provide the primary physical confinement for CO_2_, their performance is significantly amplified by surface chemistry. In ultramicropores with widths comparable to the kinetic diameter of CO_2_ (∼0.33 nm), overlapping adsorption potentials from opposing pore walls generate strong van der Waals and electrostatic fields, leading to highly efficient pore filling and preferential stabilization of CO_2_. This confinement effect is further enhanced when heteroatom‐derived basic sites are located inside these narrow pores, because spatial restriction increases the local electric field and charge density, thereby strengthening CO_2_–surface interactions. Oxygen‐, nitrogen‐, and sulfur‐containing functionalities increase surface polarity and create Lewis basic or electron‐rich sites that interact strongly with the acidic and quadrupolar CO_2_ molecules. Pyridine N and pyrrole N donate lone‐pair electrons to the carbon atom of CO_2_, while carbonyl (C═O), hydroxyl, and oxidized sulfur groups generate localized dipoles that stabilize CO_2_ through dipole–quadrupole and acid–base interactions. These interactions increase the *Q*_st_, which quantitatively reflects surface basicity and adsorption strength. Demir et al. demonstrated that heteroatom‐rich lignin‐derived carbons containing 2.5–5.6 wt% N and approximately 54 wt% O achieved a CO_2_ adsorption capacity of 4.8 mmol/g at 298 K, highlighting that heteroatom doping synergizes with microporous frameworks to maximize adsorption performance [87]. Liu et al. reported that while narrow micropores primarily govern CO_2_ adsorption capacity, higher oxygen content significantly enhances CO_2_/N_2_ selectivity and increases *Q*_st_, confirming that surface basicity directly controls adsorption affinity and selectivity [68]. Feng et al. further revealed that sulfur doping not only introduces additional polar adsorption sites but also promotes the transformation of quaternary N into pyridinic N, thereby increasing the number of basic sites and strengthening the overall CO_2_ binding energy [39]. These studies demonstrate that CO_2_ capture in lignin‐derived porous carbons is governed by a dual mechanism: ultramicropores provide size‐matched confinement that drives high CO_2_ capacity through pore filling, while N‐, O‐, and S‐containing functional groups modulate surface basicity and electrostatic interactions, thereby enhancing adsorption enthalpy, selectivity, and low‐pressure performance. Consequently, neither porosity nor heteroatom content alone fully determines CO_2_ uptake; instead, optimal adsorption results from the synergy between abundant ultramicropores and well‐balanced N, O, and S surface functionalities.

Finally, the dominance of micropores, especially ultramicropores, also explains the high CO_2_/N_2_ selectivity reported for lignin‐derived porous carbons, which is a critical parameter for flue gas separation. Comparative studies on Kraft lignin derived carbons demonstrated that samples produced under mild activation conditions (≈600 °C, KOH/carbon ratio of 1) exhibited IAST‐predicted CO_2_/N_2_ selectivity in the range of 22–38 under 15% CO_2_/85% N_2_ mixture at 25°C. This high selectivity was attributed to the prevalence of ultramicropores <0.5 nm, which strongly favor CO_2_ over N_2_ adsorption due to size exclusion and enhanced quadrupole interactions [54]. Even more remarkably, mechanochemically assisted enzymatic‐hydrolysis‐lignin carbons displayed extraordinarily high separation factors, with the Henry's law and IAST selectivity values reached 29 and 525 at 273 K and 1 bar, respectively. These exceptional values were explained by a synergistic mechanism in which ultramicroporous confinement provides strong physical adsorption, while N‐ and O‐containing functional groups further promote preferential CO_2_ binding [68]. These studies demonstrate that high CO_2_/N_2_ selectivity in lignin‐derived porous carbons is not incidental but arises directly from the interplay of ultramicropore‐dominated architectures with surface functionalities, both of which enhance preferential CO_2_ adsorption under realistic flue gas conditions.

Overall, the variability in CO_2_ adsorption performance among lignin‐derived porous carbons can be rationalized through a unified mechanistic framework that explicitly links synthesis conditions, pore structure evolution, and adsorption energetics. Chemical activation strategies and carbonization parameters primarily determine the formation or destruction of ultramicropores (<0.7 nm). These very narrow pores strongly confine CO_2_ molecules, creating overlapping interaction potentials that dominate CO_2_ uptake under post‐combustion conditions. This confinement effect is directly reflected in the isosteric heat of adsorption (*Q*_st_), which typically increases with decreasing pore width due to enhanced van der Waals and quadrupole–dipole interactions between CO_2_ molecules and the pore walls. Carbons enriched with ultramicropores therefore exhibit relatively high initial *Q*_st_ values (generally 20–40 kJ/mol), indicating strong yet reversible physisorption that is particularly effective at low CO_2_ partial pressures. In contrast, excessive activation severity or high carbonization temperatures promote pore widening and merging, shifting the pore population toward wider micropores and mesopores. Although such structures improve gas diffusion, they reduce confinement strength, leading to lower adsorption potentials, diminished *Q*_st_ values, and consequently reduced CO_2_ uptake at low pressures. As a result, CO_2_ adsorption does not scale simply with total surface area or total pore volume, but is instead governed by the fraction and accessibility of ultramicropores that provide optimal adsorption energetics. Surface heteroatom functionalities (e.g., N, O, and S species) further modulate CO_2_ affinity by increasing surface polarity and local electrostatic interactions; however, their contribution is maximized only when these functional groups are located within ultramicroporous environments that amplify interaction strength. This synergy between ultramicropore confinement and surface chemistry enables high adsorption capacity, elevated CO_2_/N_2_ selectivity, and moderate *Q*_st_ values that balance strong CO_2_ binding with energy‐efficient regeneration. Consequently, effective CO_2_ capture using lignin‐derived porous carbons arises from the coupled optimization of pore size distribution, adsorption energetics, and surface functionality, rather than from any single structural parameter alone.

### 4.2 Role of Mesopores and Hierarchical Pore Networks

**FIGURE 12:**
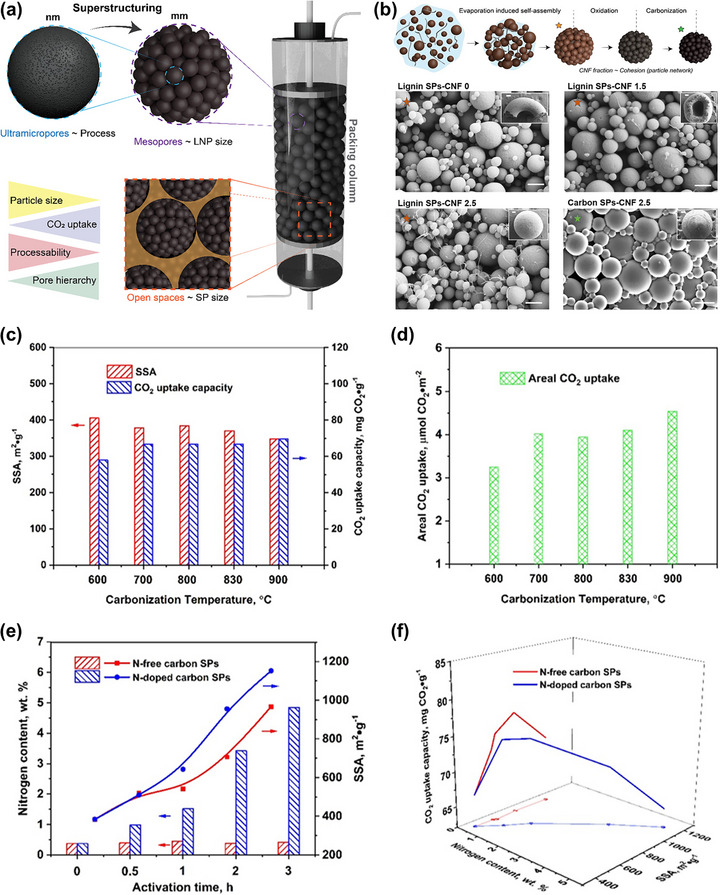
(a) Schematic illustration showing the hierarchical assembly of lignin nanoparticles into millimeter‐scaled carbon supraparticles designed for CO_2_ capture. (b) Formation pathway of carbon supraparticles through evaporation‐induced self‐assembly followed by oxidation and carbonization, SEM images of lignin‐based supraparticles containing different CNF contents (insets: lower magnification). (c) Relationship between specific surface area and CO_2_ uptake capacity, and (d) corresponding areal CO_2_ uptake of carbon supraparticles obtained at different carbonization temperatures. (e) Variation of nitrogen content and specific surface area, and (f) CO_2_ uptake performance of carbon supraparticles (carbonized at 800°C) as a function of nitrogen content and specific surface area for samples activated by steam (N‐free carbon supraparticles) or ammonia–steam (N‐doped carbon supraparticles). Reproduced with permission [98].

While micropores, particularly ultramicropores, provide the primary adsorption sites for CO_2_ at low partial pressures, mesopores (2–50 nm) and hierarchical pore networks play a vital complementary role by facilitating rapid mass transfer and ensuring efficient utilization of the microporous regions. Mesopores serve as “transport highways”, reducing diffusion resistance and allowing CO_2_ molecules to access ultramicropores more effectively, especially under dynamic or high flow rate conditions typical of post‐combustion capture. Numerous studies have shown that materials combining a high proportion of micropores with a secondary mesoporous network exhibit improved adsorption kinetics and better performance in breakthrough experiments compared to purely microporous carbons. For instance, Zhao et al. synthesized porous carbon from sodium lignosulfonate and found that the KOH‐activated carbon (**C‐LS‐KOH**) achieved the highest surface area (1998.4 m^2^/g) and micropore volume (0.7446 cm^3^/g), confirming the dominant role of micropores in CO_2_ uptake [83]. In contrast, template‐derived carbons such as **C‐LS‐MgO**, with mesopore volume exceeding 70% of the total pore volume, provided hierarchical pore networks that acted as efficient transport channels, reducing diffusion resistance and improving accessibility to micropores. Notably, the **C‐LS‐ZnCl_2_** sample, which balanced both micropores (0.5952 cm^3^/g) and mesopores (0.3133 cm^3^/g), exhibited a high CO_2_ uptake of 4.45 mmol/g at 273 K and 100 kPa, highlighting how combined micro–mesopores can optimize both adsorption capacity and mass transport efficiency. In another study, Zhao et al. developed multi‐scale carbon supraparticles by integrating lignin nanoparticles and microbeads with cellulose nanofibers (CNFs) through a soft‐templating strategy (Figure 12a) [98]. Lignin supraparticles were first assembled via evaporation‐induced self‐assembly of lignin‐CNF suspensions, where CNFs served as both a soft template and structural binder. The morphology evolution of lignin supraparticles and the resulting carbon supraparticles prepared with different CNF loadings (up to 2.5 wt%) was investigated (Figure 12b). The CO_2_ adsorption performance was then evaluated as a function of specific surface area, carbonization temperature, and nitrogen content (Figure 12c–f). The optimized lignin‐derived carbon supraparticles with hierarchical micro–mesoporous architectures achieved a CO_2_ uptake of 77 mg/g (∼1.75 mmol/g) at 40°C and 1 bar, while also presenting a low pressure drop of ∼33 kPa/m in packed‐bed simulations. The authors indicate that mesopores facilitated rapid gas diffusion and ensured full utilization of ultramicroporous adsorption sites, overcoming the mass transfer limitations typically observed in purely microporous carbons. These results indicate that mesopores and hierarchical pore networks do not directly govern equilibrium CO_2_ adsorption capacity at low pressures, but instead play a critical supporting role in determining practical adsorption performance. Their contribution lies primarily in facilitating mass transfer, accelerating adsorption kinetics, improving accessibility to ultramicroporous adsorption sites, and maintaining stable cyclic operation under realistic flow conditions. To clarify the underlying origin of this behavior, the mechanistic role of mesopores and hierarchical architectures is discussed in detail as follows. From a mechanistic perspective, the variability in CO_2_ adsorption performance associated with mesopores and hierarchical pore networks arises from their indirect but essential role in regulating mass transport and adsorption site accessibility. Mesopores (2–50 nm) do not provide strong confinement for CO_2_ molecules and therefore contribute minimally to equilibrium adsorption capacity at low pressures. Instead, their primary function is to reduce diffusion resistance and shorten transport pathways, enabling rapid and complete access of CO_2_ molecules to ultramicroporous adsorption sites. Synthesis routes that strongly favor mesopore formation (e.g., template methods) often sacrifice ultramicropore density, leading to lower intrinsic adsorption capacity despite improved kinetics. Conversely, purely chemical activation routes maximize ultramicroporosity but may suffer from slow diffusion and incomplete pore utilization under dynamic conditions. Hierarchical pore architectures emerge as an optimal compromise, in which mesopores act as transport channels that enhance the effective utilization of ultramicropores without diluting confinement‐driven adsorption potential. This explains why materials with balanced micro–mesoporous structures frequently outperform purely microporous carbons in breakthrough experiments and cyclic operation, even when their equilibrium CO_2_ uptake is similar. Therefore, the role of mesopores is not to increase CO_2_ binding strength, but to ensure that adsorption energetics governed by ultramicropores can be fully and rapidly exploited under realistic operating conditions.

### 4.3 Influence Specific Surface Area and Pore Volume

The specific surface area and pore volume of lignin‐derived porous carbons are fundamental parameters governing their CO_2_ adsorption performance. A high specific surface area generally implies more accessible adsorption sites, whereas an optimized distribution of pore volume ensures that these sites are effectively utilized under practical conditions. However, the relationship is not strictly linear; instead, CO_2_ uptake is influenced by a synergy of specific surface area, pore size distribution, and surface chemistry. For instance, Bai et al. reported N, S co‐doped carbon derived from lignin, in which increasing the KOH activation ratio greatly increased specific surface area from ∼1200 to 2100 m^2^/g and total pore volume from 0.7 to 1.4 cm^3^/g. Interestingly, the highest CO_2_ uptake (6.87 mmol/g at 273 K, 1 bar) was not obtained in the sample with the largest specific surface area, but rather in one with an optimal micropore‐to‐mesopore ratio. The authors emphasized that mesopores enhance diffusion and pore accessibility, but excessive mesoporosity diminishes the contribution of ultramicropores, thereby reducing low‐pressure affinity [27]. In the study by Dong et al., KOH‐activated and urea‐doped carbon exhibited specific surface area exceeding 1900 m^2^/g, but correlation analysis revealed that CO_2_ uptake at 298 K correlated most strongly with pore volume in the 0.6–0.8 nm range, not with total specific surface area or total pore volume. The sample with the highest specific surface area did not achieve the best CO_2_ uptake because a significant fraction of pores were larger than 1 nm and provided insufficient confinement [53]. Liu et al. further demonstrated that mechanochemically processed lignin‐derived carbons with specific surface areas ranging from 602 to 2030 m^2^/g showed no linear correlation with CO_2_ capacity. The best‐performing sample (5.00 mmol/g at 298 K, 1 bar) corresponded to an intermediate specific surface area but possessed the largest ultramicropore volume along with a high oxygen content, highlighting the synergistic effect of ultramicropores and heteroatom functionalities [68]. Similarly, Bao et al. found that urea‐modified carbons with similar textural properties but higher nitrogen content exhibited significantly greater CO_2_ uptake (3.80 mmol/g at 298 K), which they attributed to the contribution of pyridinic and graphitic nitrogen sites that strengthened CO_2_ binding [38]. These studies demonstrate that global textural parameters such as specific surface area and total pore volume alone cannot rationalize CO_2_ adsorption performance, indicating that a deeper mechanistic interpretation of how surface area and pore volume are distributed across different pore environments is required. From a mechanistic standpoint, the apparent inconsistency between specific surface area, total pore volume, and CO_2_ adsorption capacity arises because these global textural parameters do not distinguish between adsorption‐active and adsorption‐inactive surfaces. Specific surface area reflects the total internal surface generated during activation, but only the fraction associated with ultramicropores provides sufficiently strong confinement to generate high adsorption potentials for CO_2_ at low pressures. Similarly, total pore volume integrates contributions from micro‐, meso‐, and macropores, even though mesopores primarily enhance mass transport rather than equilibrium adsorption. Synthesis procedures that aggressively increase surface area (e.g., high KOH ratios or high activation temperatures) often promote pore widening and the formation of non‐confining pores, leading to high surface areas that are energetically inefficient for CO_2_ capture. In contrast, milder or more controlled activation routes preserve a higher proportion of ultramicroporous surface area, resulting in stronger CO_2_ affinity despite lower overall surface area. These observations explain why CO_2_ uptake frequently correlates poorly with total surface area or pore volume, but strongly with the volume and surface contribution of ultramicropores coupled with appropriate surface chemistry. Therefore, adsorption performance is governed not by how much surface is created, but by how that surface is distributed across energetically favorable pore environments defined by the synthesis route.

### 4.4 Amine Functionalization

Amine functionalization has emerged as an effective strategy to enhance the CO_2_ capture performance of lignin‐derived porous carbons by introducing strong chemisorptive sites that complement physisorption in micropores. The incorporation of nitrogen‐rich groups, either through impregnation of polyamines such as polyethyleneimine (PEI) or covalent grafting of alkylamines, provides additional binding affinity for acidic CO_2_ molecules via acid‐base interactions. This approach directly addresses the limitations of purely physisorptive carbons, which often suffer from low selectivity at dilute CO_2_ concentrations and reduced performance under practical operating conditions. Primary and secondary amines react with CO_2_ to form carbamate species (─NHCOO^−^) via a zwitterionic intermediate, as described by the reaction:

(1) $$2\mathrm{RN}H_{2}+{\mathrm{CO}}_{2}↔{\mathrm{RNH}}_{3}^{+}+{\mathrm{RNHCOO}}^{-}$$

In the presence of moisture, tertiary amines can capture CO_2_ by generating bicarbonate ions (HCO_3_^−^) through the reaction:

(2) $$R_{3}N+{\mathrm{CO}}_{2}+H_{2}O↔R_{3}{\mathrm{NH}}^{+}+\mathrm{HC}{O_{3}}^{-}$$

These chemical interactions not only provide high selectivity and affinity toward CO_2_ but also enhance adsorption under low partial pressures. Furthermore, the incorporation of amine groups increases the surface polarity and basicity of the carbon framework, thereby strengthening the electrostatic interactions between CO_2_ and the adsorbent. The synergistic contribution of chemical bonding on amine sites and physical adsorption within micropores accounts for the high CO_2_ adsorption performance of amine‐functionalized lignin‐derived porous carbons.

One early study by Atta‐Obeng et al. investigated hydrothermal carbons from technical lignin activated with KOH and subsequently functionalized with PEI [95]. Activation increased the specific surface area from 2.8 to 1341 m^2^/g, while optimal PEI loading (5 wt%) enhanced CO_2_ uptake from 1.53 mmol/g for the unmodified carbon to 2.0 mmol/g at 30°C. Excessive PEI (>10 wt%) reduced performance due to pore blockage, underlining the importance of balancing pore accessibility with amine content. The study demonstrated that moderate amine impregnation synergizes with microporosity to achieve capacities close to the practical benchmark of 2 mmol/g at ambient conditions. Building on this concept, Sani et al. developed mesoporous cellular carbons (MCCs) from lignin via templating, which were then impregnated with PEI [86]. The MCCs exhibited a 3D interconnected mesoporous network with high pore volume (up to 1.80 cm^3^/g) and pore size around 19 nm, enabling efficient dispersion of PEI and minimizing diffusion resistance. The resulting sorbents achieved CO_2_ adsorption capacities of 2.90–3.13 mmol/g under 15% CO_2_/N_2_ at 75–90°C. These values are significantly higher than those of conventional PEI–carbon systems. Moreover, cyclic adsorption–desorption experiments confirmed excellent thermal stability and regenerability, highlighting the suitability of these sorbents for post‐combustion capture applications. This study emphasizes that mesoporous architecture is critical for achieving high amine accessibility and stable CO_2_ uptake during repeated cycles.

**FIGURE 13:**
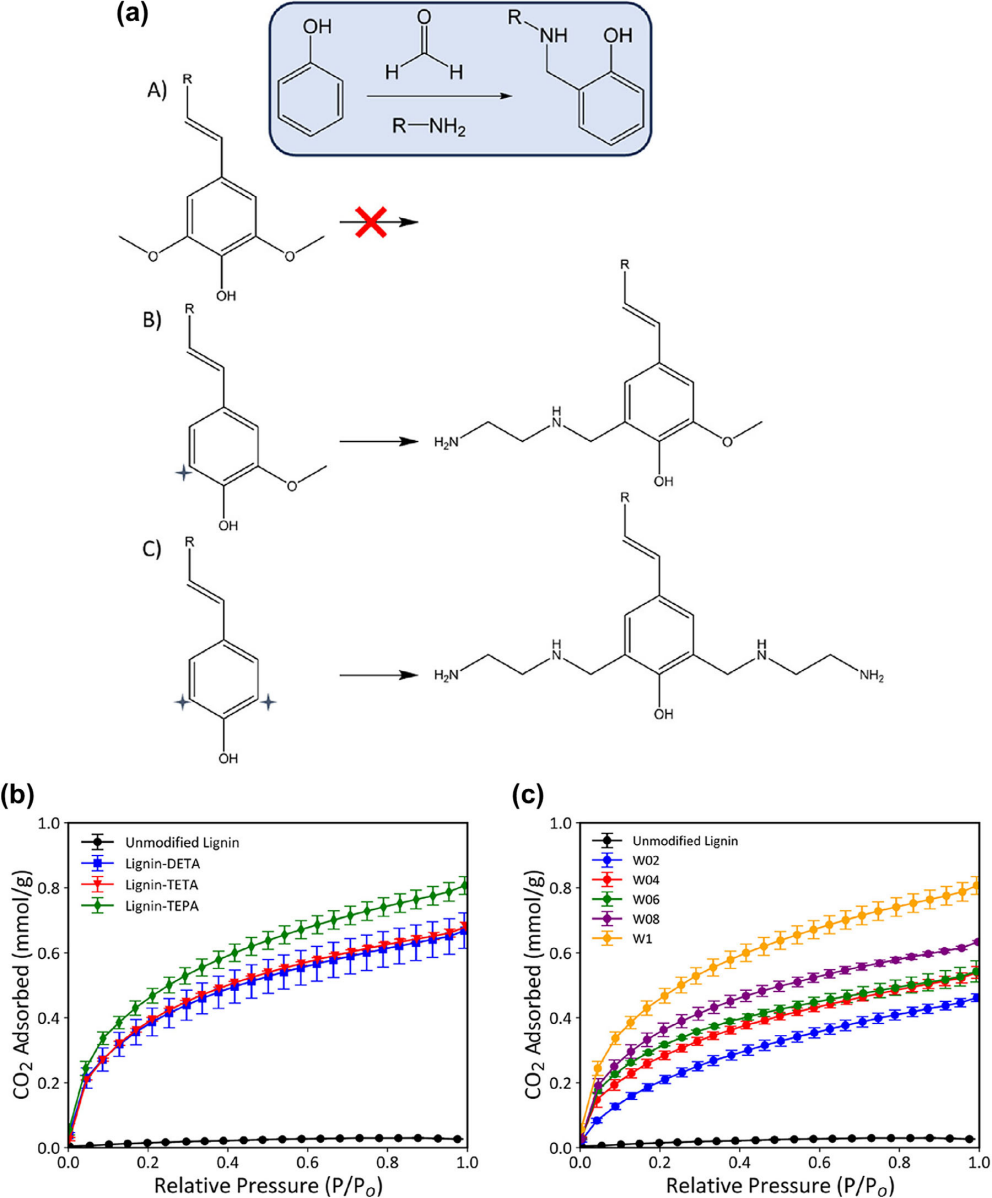
(a) General scheme showing the addition of alkylamine to coniferyl (B) and *p*‐coumaryl (C) using ethylenediamine as an example. Inset: Mannich reaction on the phenolic ring. CO_2_ adsorption isotherms of (b) unmodified lignin and aminated lignin sorbents and (c) lignin modified with different densities of TEPA. Reproduced with permission [99].

More recently, Carrier et al. demonstrated a different route by covalently grafting linear polyamines (diethylenetriamine (DETA), triethylenetetramine (TETA), and tetraethylenepentamine (TEPA)) onto industrial Kraft lignin via a one‐pot Mannich reaction (Figure 13a) [99]. Unlike conventional physical impregnation, this grafting method anchored the amine groups directly to the lignin backbone, producing stable solid sorbents with tunable amine density. The optimized material, prepared using 60 wt% TEPA, exhibited the highest CO_2_ uptake of ∼0.80 mmol/g under direct air capture (DAC) conditions (298 K, 400 ppm CO_2_) (Figure 13b,c), demonstrating that covalent functionalization can produce sorbents tailored for ultra‐dilute CO_2_ capture. Although the uptake is lower than that achieved under concentrated flue gas conditions, the performance is notable given the extremely low CO_2_ partial pressure in DAC applications. The study further highlighted that sorbent modified with longer‐chain TEPA molecules outperformed those with shorter amines (DETA and TETA), attributed to both the higher density of secondary amine sites and greater molecular flexibility of TEPA, which facilitates proton transfer and stabilizes carbamate intermediates. These findings demonstrate that covalent functionalization of lignin with flexible polyamines can yield low‐cost, regenerable sorbents optimized for atmospheric CO_2_ capture applications.

These studies show that the amine functionalization of lignin‐derived carbons can significantly improve both capacity and selectivity, but the mechanism depends strongly on the functionalization route and pore structure. From a unified mechanistic standpoint, amine functionalization introduces a fundamentally different adsorption regime in lignin‐derived porous carbons by partially decoupling CO_2_ uptake from ultramicropore‐controlled physisorption. Unlike purely physical adsorption, which depends strongly on pore confinement and adsorption energetics, chemisorption on amine sites is governed by the accessibility, dispersion, and chemical environment of the amine functionalities. As a result, pore architecture plays an indirect but critical role by determining whether amine groups remain accessible or become diffusion‐limited or sterically hindered. In systems based on physical impregnation, excessive amine loading or insufficient mesoporosity leads to pore blockage and reduced performance, whereas hierarchical mesoporous networks facilitate uniform amine distribution and rapid CO_2_ transport. In contrast, covalent grafting strategies minimize amine migration and leaching, enabling stable CO_2_ capture even under ultra‐dilute conditions, although often at lower gravimetric capacities due to reduced amine density. These differences explain the wide variability in reported CO_2_ uptake among amine‐functionalized lignin‐derived carbons and demonstrate that adsorption performance is dictated not solely by amine content, but by the interplay between functionalization route, pore architecture, and mass transport. Consequently, effective amine‐based CO_2_ capture requires pore structures specifically tailored to support chemisorption, rather than those optimized solely for physisorption.

## 5 Artificial Intelligence‐Assisted Design of Lignin‐Derived Porous Carbons For CO_2_ Capture

In recent years, the rapid development of artificial intelligence (AI) and machine learning (ML) has opened new opportunities for designing porous carbons with enhanced CO_2_ capture performance. Unlike traditional trial‐and‐error synthesis or purely physics‐based modeling, AI‐driven strategies rely on compiling large datasets of materials’ structures, properties, and operating conditions, which are then analyzed using ML algorithms to identify hidden correlations and predict optimal performance. The key advantage of this approach is its ability to process complex, nonlinear relationships between synthesis parameters, pore structures, surface chemistry, and CO_2_ adsorption behavior that are difficult to capture with conventional models.

**FIGURE 14:**
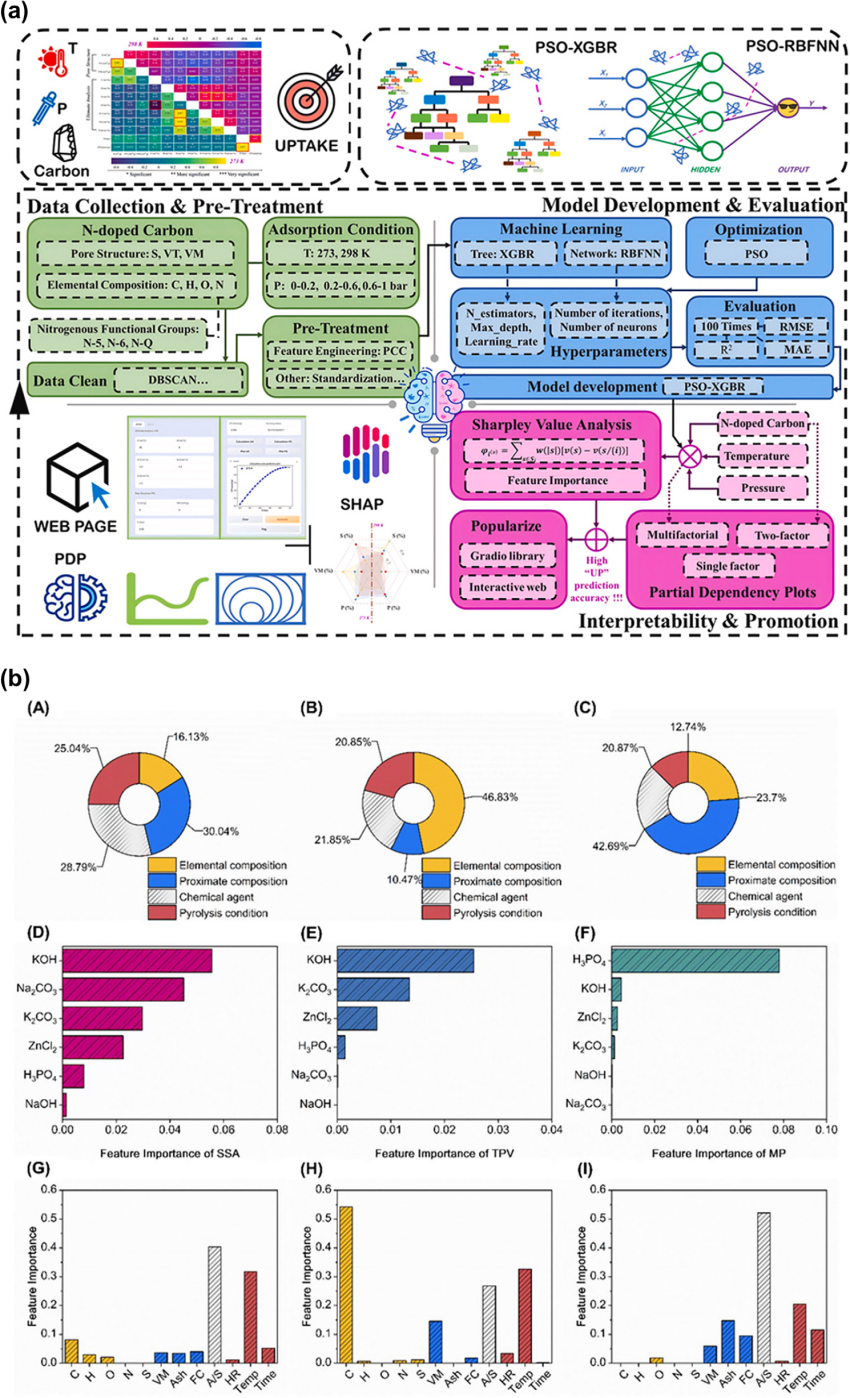
(a) Machine learning models (PSO‐XGBR and PSO‐RBFNN) were developed to predict CO_2_ adsorption behavior of N‐doped porous carbons under variable temperature and pressure conditions. The integration of SHAP and PDP analyses revealed that micropore volume and pyridinic/pyrrolic nitrogen contents are the most influential parameters governing CO_2_ uptake, while the optimized models achieved high predictive accuracy (*R* ^2^ = 0.80–0.97). This study provides a data‐driven strategy for designing high‐performance carbon adsorbents and enables rapid prediction through an interactive web‐based platform. Reproduced with permission [101]. (b) Contribution of individual input features to specific surface area, total pore volume, and microporosity using the Random Forest (RF)‐based Gradient Boosting Regression (GBR) model. (A–C): feature contribution of each step, (D–F): feature contribution of different chemical activators, (G–I): feature contribution of other features under the catalytic pyrolysis of KOH or H_3_PO_4_. Reproduced with permission [102].

For lignin‐derived porous carbons, current research is moving from exploratory demonstrations toward more systematic integration of AI. It should be emphasized that, to date, only a very limited number of studies have explicitly applied ML to lignin‐derived porous carbons for CO_2_ capture. Consequently, AI‐assisted design of lignin‐derived adsorbents for CO_2_ capture remains at an early stage of development. Recent work by Khoshraftar and Ghaemi represents a significant step toward data‐driven design of nitrogen‐doped porous carbon derived from lignin for CO_2_ capture [100]. By integrating isotherm modeling, ML, and response surface methodology, they established a quantitative link between pore architecture, operating conditions, and adsorption performance. Their analysis revealed that although specific surface area and total pore volume define the maximum storage capacity, CO_2_ uptake is predominantly activated by pressure‐driven filling of ultramicropores enriched with nitrogen functionalities. Random forest modeling showed that pressure is the dominant variable governing adsorption, while response surface methodology optimization demonstrated that optimal performance is achieved only when surface area and pore volume are properly balanced, rather than maximized independently. These findings indicate that CO_2_ adsorption in lignin‐derived carbons follows a cooperative, pore‐filling mechanism controlled by hierarchical structural features, and that ML can be used not merely for prediction but as a powerful tool to uncover the governing structure‐property‐process relationships. More recently, Wang et al. developed a comprehensive ML framework to decode CO_2_ adsorption in nitrogen‐doped porous carbons across variable temperature and pressure conditions (Figure 14a) [101]. By integrating XGBoost models with SHapley Additive exPlanations (SHAP) and partial‐dependence analysis on a dataset of over 1000 literature samples, they demonstrated that pore structure, particularly ultramicropore volume, governs CO_2_ uptake more strongly than elemental composition. At low pressures, adsorption is dominated by micropore filling reinforced by pyridinic and pyrrolic nitrogen sites, whereas at higher pressures multilayer adsorption becomes increasingly important. Crucially, multivariate analysis revealed a narrow optimal design window, where a micropore volume above 0.6 cm^3^/g combined with 3.5–4.5 wt% pyridinic nitrogen yields CO_2_ uptakes exceeding 3 mmol/g. This work shows that AI can move beyond prediction to provide physically interpretable design rules, enabling rational tuning of nitrogen‐doped lignin‐derived carbons for efficient and regenerable CO_2_ capture.

In another direction, Xie et al. developed a hybrid ML framework to quantitatively predict the pore structure of lignin‐derived porous carbons produced by catalytic pyrolysis across six chemical activators (KOH, ZnCl_2_, H_3_PO_4_, K_2_CO_3_, NaOH, and Na_2_CO_3_) (Figure 14b) [102]. By combining a Random Forest‐based data‐interpolation model with a Gradient Boosting regressor, they overcame the long‐standing problem of incomplete literature datasets and achieved accurate predictions of specific surface area, total pore volume, and microporosity (R^2^ up to 0.86). Feature‐importance and partial‐dependence analyses revealed that chemical activators dominate pore formation, with KOH controlling high surface area and pore volume through aggressive carbon etching, while H_3_PO_4_ governs micropore generation via dehydration‐driven framework restructuring. Importantly, the model captured nonlinear “volcano‐type” relationships between activator‐to‐lignin ratio, temperature, and pore development, providing quantitative guidelines for selecting optimal activation conditions prior to experimentation. This work demonstrates how ML can be used not merely for property prediction, but as a mechanistic tool to rationally design lignin‐derived carbons with targeted pore architectures. Building on this activator‐guided pore engineering framework, the same group further demonstrated that the predictive power of ML for lignin‐derived porous carbons is fundamentally limited not by data volume but by data quality. By introducing a feature‐engineering‐based hybrid ML approach, they algorithmically generated over 2000 physically meaningful descriptors from existing lignin composition and pyrolysis parameters, selecting 30 high‐correlation features to enrich the dataset [103]. This dramatically improved the performance and generalizability of XGBoost models, achieving R^2^ values of 0.85 for specific surface area and 0.89 for total pore volume. Importantly, SHAP and partial‐dependence analysis showed that the generated features captured real physicochemical mechanisms, revealing how lignin carbon content, volatile matter, and carbonization temperature govern framework rigidity, gas‐induced pore nucleation, and mesopore expansion. Together with their earlier activator‐screening model, this work establishes a data‐driven digital twin for catalytic pyrolysis of lignin, enabling rational, experiment‐free tuning of nanopore architectures for high‐performance adsorption applications.

These studies demonstrate that AI can accelerate the design of lignin‐derived porous carbons by bridging synthesis parameters, pore structures, and adsorption performance. However, progress is still limited by the availability and quality of reliable datasets. Most reported models are trained on relatively small collections of experimental results, and often lack detailed descriptors such as ultramicropore distribution or surface functional group speciation. Building a more comprehensive database, including lignin source, extraction method, processing conditions, and adsorption measurements, will be crucial for enabling robust and generalizable AI models. In parallel, combining experimental data with molecular simulations and computational chemistry can provide valuable complementary datasets, helping to train ML models at lower cost. While AI applications in lignin‐derived porous carbons are still emerging, the early successes clearly demonstrate their potential to accelerate discovery, optimize synthesis, and identify key design rules for CO_2_ capture. The integration of data‐driven methods with experimental and simulation approaches will be essential to move from correlation‐based screening toward predictive, inverse design of next‐generation sustainable sorbents.

## 6 Conclusion and Outlook

Lignin‐derived porous carbons have emerged as one of the most promising classes of sustainable sorbents for CO_2_ capture. Their abundance, high carbon content, and intrinsic aromatic backbone enable the production of porous carbons with tunable textural and surface properties that rival or exceed those of many synthetic adsorbents. This review highlights how advances in chemical activation, templating, multistep processing, and surface functionalization have enabled precise control over pore size distribution, ultramicroporosity, and heteroatom incorporation, all of which are decisive factors for adsorption capacity, selectivity, and regeneration energy. Particular emphasis has been placed on the critical role of ultramicropores (<0.7 nm) in low‐pressure CO_2_ uptake, the complementary function of mesopores in enhancing mass transfer, and the synergistic effects of nitrogen, sulfur, or oxygen functionalities in strengthening CO_2_ affinity while maintaining energy‐efficient regeneration. Emerging strategies such as amine impregnation and covalent amine grafting further extend the operating window toward post‐combustion and even direct air capture conditions by introducing strong chemisorptive binding sites.

Despite these advances, several challenges remain before lignin‐derived carbons can be deployed at scale. The intrinsic heterogeneity of lignin, arising from plant species, extraction method, and pulping history, continues to introduce variability in carbon yield, pore structure, and surface chemistry, complicating process standardization and reproducibility. Many high‐performance activation routes still rely on corrosive chemicals (e.g., KOH) or multi‐step treatments that increase cost and environmental burden. Achieving simultaneous control of ultramicropore volume, hierarchical pore connectivity, and stable heteroatom doping in a single scalable process remains a key materials challenge. Furthermore, techno‐economic assessments and life‐cycle analyses are still scarce but are essential to benchmark lignin‐derived carbons against alternative adsorbents such as MOFs, COFs, and advanced membranes.

Looking forward, several directions appear particularly promising.

First, greener activation strategies, such as potassium carbonate activation, spray drying‐assisted carbonization, should be further optimized to reduce chemical usage and improve yield while maintaining high ultramicroporosity.

Second, a deeper mechanistic understanding of lignin carbonization, including in situ characterization of pore evolution and heteroatom migration, will enable rational bottom‐up design of pore structures and surface chemistries rather than empirical screening.

Third, beyond material innovation, integration with process‐level and techno‐economic assessments is essential to bridge laboratory performance with practical deployment. Incorporating process simulations such as pressure–swing or temperature–swing adsorption (PSA/TSA), cyclic stability tests under humid or mixed‐gas conditions, and life‐cycle carbon footprint assessments can establish realistic performance benchmarks. Such multiscale evaluation will clarify the competitiveness of lignin‐derived carbons against other solid sorbents (e.g., MOFs, amine‐functionalized silica) and guide engineering design toward modular, low‐cost CO_2_ capture systems.

Finally, the integration of artificial intelligence and machine learning offers a transformative path for data‐driven materials discovery. Recent studies already show that machine learning models can identify key synthesis parameters, predict textural properties, and even provide interpretable design rules linking nitrogen content, ultramicropore volume, and CO_2_ uptake. Expanding high‐quality experimental databases and coupling them with molecular simulations will be crucial to realizing predictive, inverse design of lignin‐derived carbons.

Overall, lignin provides a unique convergence of sustainability, abundance, and chemical versatility for next‐generation CO_2_ capture materials. By combining greener synthesis routes, advanced characterization, and artificial intelligence‐assisted design, future research can overcome the current barriers of feedstock variability, process scalability, and performance reproducibility. Continued progress along these lines will accelerate the deployment of lignin‐derived porous carbons as practical, circular, and economically viable solutions for large‐scale carbon capture.

## Conflicts of Interest

The authors declare no conflicts of interest.
